# *N* ^2^-methylguanosine modifications on human tRNAs and snRNA U6 are important for cell proliferation, protein translation and pre-mRNA splicing

**DOI:** 10.1093/nar/gkad487

**Published:** 2023-06-07

**Authors:** Can Wang, Nathalie Ulryck, Lydia Herzel, Nicolas Pythoud, Nicole Kleiber, Vincent Guérineau, Vincent Jactel, Chloé Moritz, Markus T Bohnsack, Christine Carapito, David Touboul, Katherine E Bohnsack, Marc Graille

**Affiliations:** Laboratoire de Biologie Structurale de la Cellule (BIOC), CNRS, École polytechnique, Institut Polytechnique de Paris, 91120 Palaiseau, France; Laboratoire de Biologie Structurale de la Cellule (BIOC), CNRS, École polytechnique, Institut Polytechnique de Paris, 91120 Palaiseau, France; Department of Molecular Biology, University Medical Center Göttingen, 37073 Göttingen, Germany; Laboratoire de Spectrométrie de Masse BioOrganique, CNRS, Université de Strasbourg, IPHC UMR 7178, Infrastructure Nationale de Protéomique ProFI, FR2048 Strasbourg, France; Department of Molecular Biology, University Medical Center Göttingen, 37073 Göttingen, Germany; Université Paris-Saclay, CNRS, Institut de Chimie des Substances Naturelles, UPR 2301, 91198 Gif-sur-Yvette, France; Laboratoire de Synthèse Organique (LSO), CNRS, École polytechnique, ENSTA, Institut Polytechnique de Paris, 91120 Palaiseau, France; Laboratoire de Spectrométrie de Masse BioOrganique, CNRS, Université de Strasbourg, IPHC UMR 7178, Infrastructure Nationale de Protéomique ProFI, FR2048 Strasbourg, France; Department of Molecular Biology, University Medical Center Göttingen, 37073 Göttingen, Germany; Cluster of Excellence “Multiscale Bioimaging: from Molecular Machines to Networks of Excitable Cells” (MBExC), Göttingen, Germany; Laboratoire de Spectrométrie de Masse BioOrganique, CNRS, Université de Strasbourg, IPHC UMR 7178, Infrastructure Nationale de Protéomique ProFI, FR2048 Strasbourg, France; Université Paris-Saclay, CNRS, Institut de Chimie des Substances Naturelles, UPR 2301, 91198 Gif-sur-Yvette, France; Laboratoire de Chimie Moléculaire (LCM), CNRS, École polytechnique, Institut Polytechnique de Paris, 91120 Palaiseau, France; Department of Molecular Biology, University Medical Center Göttingen, 37073 Göttingen, Germany; Laboratoire de Biologie Structurale de la Cellule (BIOC), CNRS, École polytechnique, Institut Polytechnique de Paris, 91120 Palaiseau, France

## Abstract

Modified nucleotides in non-coding RNAs, such as tRNAs and snRNAs, represent an important layer of gene expression regulation through their ability to fine-tune mRNA maturation and translation. Dysregulation of such modifications and the enzymes installing them have been linked to various human pathologies including neurodevelopmental disorders and cancers. Several methyltransferases (MTases) are regulated allosterically by human TRMT112 (Trm112 in *Saccharomyces cerevisiae*), but the interactome of this regulator and targets of its interacting MTases remain incompletely characterized. Here, we have investigated the interaction network of human TRMT112 in intact cells and identify three poorly characterized putative MTases (TRMT11, THUMPD3 and THUMPD2) as direct partners. We demonstrate that these three proteins are active *N*^2^-methylguanosine (m^2^G) MTases and that TRMT11 and THUMPD3 methylate positions 10 and 6 of tRNAs, respectively. For THUMPD2, we discovered that it directly associates with the U6 snRNA, a core component of the catalytic spliceosome, and is required for the formation of m^2^G, the last ‘orphan’ modification in U6 snRNA. Furthermore, our data reveal the combined importance of TRMT11 and THUMPD3 for optimal protein synthesis and cell proliferation as well as a role for THUMPD2 in fine-tuning pre-mRNA splicing.

## Graphical Abstract

**Graphical Abstract:**
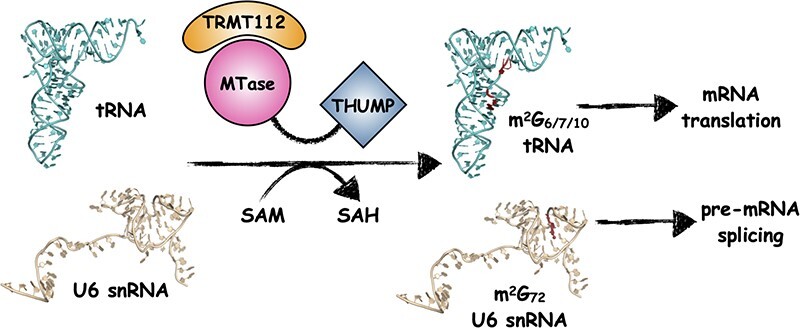

## INTRODUCTION

In recent years, the epitranscriptome, a term collectively describing modified nucleotides throughout the transcriptome, has emerged as a key aspect of eukaryotic gene expression regulation (1). Indeed, almost all RNA types involved in splicing (small nuclear (sn)RNAs) or translation (transfer (t)RNAs and ribosomal (r)RNAs) of messenger (m)RNAs are decorated with numerous chemically diverse modifications often referred to as epitranscriptomic marks (2–6). These marks are often conserved during evolution but many of their functions have remained elusive due to the lack of obvious phenotypes or because they are ‘orphan’ modifications for which the cognate writer enzymes remain unknown. Importantly, as writer enzymes are progressively identified, the critical roles of these RNA modifications in cellular development and in human pathologies, such as neurodevelopmental disorders and cancers, are being elucidated (4,7–15).

The most frequent RNA modification is methylation. It is primarily catalyzed by *S*-adenosyl-l-methionine (SAM)-dependent MTases, which structurally belong to two main classes: class I enzymes adopting a Rossmann fold and class IV with a SPOUT trefoil knotted fold (16). In eukaryotes, many class I enzymes are active as holoenzymes composed of a catalytic subunit associated with at least one activating partner (17,18). A prominent example is METTL3-METTL14, the major m^6^A mRNA MTase complex (19) but it is also the case for many tRNA MTases, such as the METTL1-WDR4 (Trm8–Trm82 in *S. cerevisiae* (yeast)), FTSJ1-WDR6 (Trm7–Trm732 in yeast) and TRMT61A-TRMT6 (Trm61–Trm6 in yeast) complexes (17,18).

Interestingly, TRMT112 proteins, which are conserved in all three domains of life, acts as an allosteric regulator of several archaeal and eukaryotic MTases targeting diverse substrates (tRNAs, rRNAs and proteins) involved in protein synthesis (17,20,21). The TRMT112 protein and its associated MTases are best characterized in *S. cerevisiae* while less is known about the human homologues. In yeast, Trm112 interacts with four MTases: Bud23 (BUD23 or WBSCR22 in human), Mtq2 (HEMK2 or MTQ2 in human), Trm9 (ALBKH8 in human) and Trm11 (probably TRMT11 in human). The Trm112–Bud23 complex catalyzes the formation of 7-methylguanosine (m^7^G) on the 18S rRNA and participates in the 40S ribosomal subunit biogenesis pathway (22,23). The Trm112–Mtq2 complex modifies the side chain of the glutamine residue of the universally conserved GGQ motif from the translation termination factor eRF1, which recognizes stop codons present in the ribosomal A-site (24,25,26,27). This eRF1 motif enters into the peptidyl transferase center of ribosomes to trigger the release of newly synthesized proteins (28). This complex has also recently been shown to be important for the biogenesis of the large ribosomal subunit (29). In humans, this complex also modifies the lysine 12 side chain of histone H4 and is important for prostate cancer cell proliferation (30). The yeast Trm112–Trm9 and Trm112–Trm11 complexes contribute to translation elongation by modifying specific tRNAs (31–35). Trm9 is responsible for adding a methyl group to 5-carboxymethyl-(2-thio)-uridine (cm^5^(s^2^)U) to form 5-methoxycarbonylmethyl-(2-thio)-uridine (mcm^5^(s^2^)U) at position 34 of some tRNAs (32–35). In humans, two proteins share significant similarities with yeast Trm9 and both interact with TRMT112: ALKBH8 modifies tRNAs in a similar manner to Trm9 (36,37) while the biochemical function of TRMT9B (also known as KIAA1456 or hTrm9L), a protein acting as a tumor suppressor, remains to be clarified (38,39). Trm11 catalyzes the formation of m^2^G at position 10 of some tRNAs in both yeast and several archaea (31,40–44). Identification and characterization of these TRMT112-containing holoenzymes have highlighted common features. For example, TRMT112 (i) stabilizes these MTases *in cellulo*, (ii) activates them by enhancing SAM binding, and (iii) contributes to substrate binding. Furthermore, the MTases share a common mode of interaction with TRMT112 such that their interactions are mutually exclusive (17,20).

In metazoans, the METTL5 MTase, a protein with no orthologue in *S. cerevisiae* yeast, was identified as a direct partner of TRMT112 and subsequently, the TRMT112–METTL5 complex was revealed to mediate the formation of *N*^6^-methyladenosine (m^6^A) on the 18S rRNA (45–50). This demonstrated that the interaction network of human TRMT112 extends beyond the homologues of the MTases known from yeast. Indeed, a recent study uncovered seven MTases (BUD23, METTL5, MTQ2, ALKBH8, TRMT11, THUMPD2 and THUMPD3) associated with TRMT112 (51) and TRMT112-THUMPD3 was shown to target specific tRNAs, where it introduces m^2^G modifications at position 6 (or position 7 in the case of tRNA^Trp^) (52).

Here, we identified human TRMT112-interacting proteins using an unbiased proximity labelling approach in intact cells, revealing not only associated MTases, but also numerous other factors. We focus on three TRMT112-MTase complexes, TRMT112-TRMT11, -THUMPD2 and -THUMPD3 complexes as the roles of the tRNA m^2^G_6/7_ modifications installed by TRMT112-THUMPD3 in translation regulation are not yet understood, TRMT11 has not yet been characterized in human cells and the function of THUMPD2 remains elusive. We characterize these three holoenzymes as m^2^G MTases modifying small RNAs and show that their absence affects the proliferation of HCT116 colon cancer cells. TRMT11 and THUMPD3 are responsible for m^2^G formation at positions 10 and 6, respectively, of specific tRNAs. We show that lack of TRMT11, THUMPD3 or both m^2^G tRNA MTases does not significantly affect substrate tRNA stability, folding or aminoacylation. However, we demonstrate that the absence of m^2^G in tRNAs impairs protein synthesis. Notably, our data indicate that m^2^G modifications at positions 6/7 and 10 function synergistically. Excitingly, we also discover that THUMPD2 binds the U6 snRNA, the central snRNA involved in the pre-mRNA splicing reaction, and is essential for m^2^G formation on this snRNA, an ‘orphan’ modification identified more than 40 years ago (5,53,54). In rodents (5,53,54), this m^2^G is found at position 72, which is located within the spliceosome catalytic core, and we reveal that this enzyme is important for pre-mRNA splicing in human cells.

## MATERIALS AND METHODS

### Statistical analyses

Unless otherwise stated, all data are expressed as mean ± standard deviation (SD) calculated from three (growth curves, enzymatic activities, AHA incorporation, RNA levels) or five (polysome profiles by sucrose gradient, m^2^G and m^2,2^G quantifications) biological replicates. Statistical differences were determined using an independent two-tailed Student's *t-*test to compare values for a given mutant to those of the controls (WT samples). **** *P* < 0.0001; *** *P* < 0.001; ** *P* < 0.01; * 0.01 < *P* < 0.05; ns: not statistically significant. Details of the statistical analyses performed during bioinformatics analyses are given in the corresponding sections below.

### Molecular cloning

Plasmids used in this study are listed in Supplementary Table S1. Plasmids generated in this study were created using standard cloning methods involving the oligonucleotides listed in Supplementary Table S2, restriction enzyme digestions and T4 ligation. The human *TRMT112* coding sequence was amplified from pFF6 (kind gift from Dr. V. Heurgué-Hamard). *In vitro* synthesized, codon optimized DNA fragments encoding the human *THUMPD2* or *THUMPD3* coding sequences (obtained from Integrated DNA Technologies, Belgium) were used as templates in the preparation of plasmids encoding for THUMPD2 or THUMPD3, respectively. For co-expression of the *Bos taurus* (*Bt*) THUMPD2–TRMT112 complex in *E. coli*, an *in vitro* synthesized DNA fragment (Integrated DNA Technologies, Belgium) containing the coding sequences of both proteins (His-tagged *Bt*THUMPD2 and untagged *Bt*TRMT112) was used as a template to generate pMG1032. The coding sequence of human *TRMT11* was amplified by PCR from the HsCD00515724 plasmid (DNASU repository, (55)). Plasmids for expression of guide RNAs for CRISPR-Cas genome editing were based on pSpCas9(BB)-2A-puro vector and were generated by cloning of annealed oligonucleotides with appropriate restriction enzyme cleavage site overhangs. To generate stably transfected HEK293 cell lines for the inducible expression of N-terminally 2 × Flag-His_6_ or C-terminally His_6_-2 × Flag tagged THUMPD2, the *THUMPD2* CDS was cloned into appropriate pcDNA5-based plasmids (Supplementary Table S1). Site-directed mutagenesis was performed according to Zheng *et al.* (56) where necessary using oligonucleotides listed in Supplementary Table S2.

### BioID (proximity-dependent biotin identification)-mass spectrometry (MS)

The BioID experiments were performed mostly as described in Chapat *et al.* (57). Briefly, 8 × 10^6^ HEK293T cells were cultured in DMEM complete medium (DMEM medium (Gibco) supplemented with 10% fetal bovine serum (Gibco), 200 U/ml of penicillin and 200 μg/ml streptomycin) in 15 cm plates at 37°C and 5% of CO_2_. 24 h after seeding or when the cells reached 80% confluence, pcDNA3.1-based plasmids for the expression of BirA(R118G)*-HA tagged eGFP (pMG1127), TRMT112 (pMG891) or TRMT112-T5R (pMG906) or the BirA*-HA cassette alone (Addgene #36047) were transiently transfected (Supplementary Table S1). For transient transfection, 5 μg of plasmids (2 μg of pcDNA3.1 MCS-BirA(R118G)-HA or of pMG1127 supplemented with 3 μg of empty pcDNA3.1 plasmid or 5 μg of pMG891 or of pMG906) in a total volume of 300 μl of Opti-MEM medium (Gibco) were mixed with 300 μl of Opti-MEM medium containing 15 μl of Lipofectamine 2000 (Invitrogen) and added gently to the plates. After 24 h, 50 μM biotin was added to the culture medium and cells were grown for a further 24 h. Cells were washed with 1xPBS (Gibco) and harvested in lysis buffer (50 mM Tris–HCl, pH 7.5, 150 mM NaCl, 1% Nonidet *P*-40, 0.4% SDS, 1.5 mM MgCl_2_, 1 mM EGTA, benzonase, and cOmplete EDTA-free Protease inhibitor (1 × PIC)). Lysates were rotated at 4°C for 30 min and then centrifuged at 16 000 × g for 20 min at 4°C. For each condition, equal amounts of proteins were incubated with anti-Streptavidin high performance affinity resin (Cytiva; #17511301) pre-equilibrated in lysis buffer and incubated overnight at 4°C. Next, the supernatant was discarded and the beads were washed once with 500 μl of SDS wash buffer (50 mM Tris⋅HCl, pH 7.5, and 2% SDS), twice with lysis buffer, three times with 50 mM ammonium bicarbonate (pH 8.0). Proteins aggregated on beads were resuspended in 100 μl of NH_4_HCO_3_ at 25 mM containing 2 μg of Trypsin/Lys-C mix (Mass Spec Grade, Promega, Madison, WI, USA). Digestion was performed under agitation at 37°C during 4 h. Then, the same enzyme amount was added to the solution and the second digestion step was conducted overnight at 37°C under agitation. Supernatant was collected after a centrifugation at 500 × g for 2 min, and beads were washed with water. Both solutions were pooled. The enzymatic digestion was stopped by adding 6 μl of pure formic acid (FA, 2% final concentration). Samples were then centrifuged at 16 000 × g for 5 min and supernatants were collected (90% of the solution). Finally, the samples were vacuum dried, and then resolubilized in 400 μl of H_2_O/ACN (acetonitrile)/FA (98/2/0.1 v/v/v) prior to nanoLC–MS/MS analysis. Each condition was performed in five replicates.

NanoLC–MS/MS analyses were performed on a nanoAcquity UPLC device (Waters Corporation, Milford, USA) coupled to a Q-Exactive HF-X mass spectrometer (Thermo Fisher Scientific, Bremen, Germany). Peptides were loaded on a symmetry C18 pre-column (20 mm × 180 μm with 5 μm diameter particles, Waters) before being separated on an ACQUITY UPLC BEH130 C18 column (250 mm × 75 μm with 1.7 μm diameter particles). The solvent system consisted of 0.1% FA in water (solvent A) and 0.1% FA in ACN (solvent B). Samples were loaded into the enrichment column over 3 min at 5 μl/min with 99% of solvent A. Peptides were then eluted at 450 nl/min with the following gradient of solvent B: from 1 to 8% over 2 min, 8 to 35% over 77 min, and 35 to 90% over 1 min. The system was operated in a data-dependent acquisition (DDA) mode with automatic switching between MS (mass range 375–1500 *m/z* with *R* = 120 000 at 200 *m/z*, automatic gain control fixed at 3 × 10^6^ ions, and a maximum injection time set at 60 ms) and MS/MS (mass range 200–2000 *m/z* with *R* = 15 000 at 200 *m/z*, automatic gain control fixed at 1.105, and the maximal injection time set to 60 ms) modes. The twenty most abundant peptides were selected on each MS spectrum for further isolation and higher energy collision dissociation (normalized collision energy set to 27), excluding unassigned, singly charged and over seven times charged ions. The dynamic exclusion time was set to 40 s.

Raw nanoLC–MS/MS data were processed using MaxQuant software (version 1.6.6.0). Peaks were assigned with the Andromeda search engine with trypsin specificity. The database used for the searches was extracted from UniProtKB-SwissProt and included all *Homo sapiens* entries (22 July 2019; Taxonomy ID = 9606; 20 409 entries). The minimum peptide length required was seven amino acids and a maximum of one missed cleavage was allowed. The precursor mass tolerance was set to 20 ppm for the first search and 4.5 ppm for the main search. The fragment ion mass tolerance was set to 20 ppm. Methionine oxidation and acetyl (Protein N-term) were set as variable modifications. The maximum false discovery rate was 1% for peptides and proteins with the use of a decoy strategy. The ‘match between runs’ option was desactivated. Unique peptides were used but modified peptides as well as their unmodified counterparts, were excluded from protein quantification. We used the ‘proteingroups.txt’ file with intensities (non-normalized intensities). The dataset was deposited to the ProteomeXchange Consortium via the PRIDE partner repository with the dataset identifier PXD038997 (58). Statistical analyses were performed using ProStaR software (59). Only proteins for which 5 intensity values were available in a single condition were kept. After log2 transformation, intensities were normalized within condition using vsn method and imputation of missing values were performed. For each sample, the slsa algorithm was used for the POV (Partially Observed Values) imputation, and missing values were replaced by the 2.5 percentile value for the MEC (Missing on the Entire Condition); statistical testing was performed using Limma. Benjamini–Hochberg method was used to adjust p-values for multiple testing and differentially expressed proteins were sorted out using a p-value threshold that guarantees an FDR below 1%.

### Co-immunoprecipitation (Co-IP) and western blotting

3.5 × 10^6^ HEK293T cells were seeded in 10 cm plates and the next day, were transfected with 2 μg of plasmids using Lipofectamine 2000 as described above. After 24 h, cells were harvested and cleared cell extracts were prepared as described above. Cell extracts were incubated overnight at 4°C with either anti-Flag M2 affinity agarose gel (Sigma-Aldrich; #A2220) or anti-HA agarose beads (Pierce™, #26181) pre-equilibrated with lysis buffer (50 mM HEPES–KOH pH 7.5, 100 mM KCl, 2 mM EDTA, 0.1% NP40, 10% glycerol, 1 mM PMSF, 1 mM DTT supplemented with 1 × PIC). After several washing steps with the wash buffer (50 mM HEPES–KOH pH 7.5, 100 mM KCl, 2 mM EDTA), the beads were mixed with 2 × loading buffer (80 mM Tris–HCl pH 6.8, 20% glycerol, 2% SDS, 0.01% bromophenol blue, 2% 2-mercaptoethanol) and eluates were separated by sodium dodecyl-sulfate polyacrylamide gel electrophoresis (SDS-PAGE) prior to transfer on nitrocellulose membrane for western blotting using antibodies described in Supplementary Table S3.

### IP-MS to detect proteins associated with THUMPD2

8 × 10^6^ HEKT293T cells were seeded in 15 cm dishes and the next day, were transiently transfected with 5 μg of plasmids (pMG976 or pMG1128; Supplementary Table S1) as described above. Cells were collected in lysis buffer (50 mM HEPES–KOH pH 7.5, 100 mM KCl, 2 mM EDTA, 0.1% NP40, 10% glycerol, 1 mM PMSF, 1mM DTT supplemented with 1 × PIC) and a freeze-thaw strategy involving freezing cells in liquid nitrogen for 5 min and thawing at 37°C was used for lysis. The lysate was then incubated at 4°C for 20 min followed by centrifugation (20 000 × g, 4°C; 15–30 min) to remove cellular debris. Cleared cell extracts were incubated with pre-washed anti-Flag beads overnight at 4°C following which, the beads were washed five times with lysis buffer complemented with 1 × PIC. The washed beads were directly frozen in liquid nitrogen and stored at -80°C. Samples were sonicated prior to transfer to 1.5 ml Eppendorf tubes compatible with a magnetic rack. After incubating the beads on the magnetic rack, the supernatant was discarded. The beads were washed three times with 200 μl of 50 mM ammonium bicarbonate and finally resuspended in 100 μl of ammonium bicarbonate (50 mM) before the addition of 2 μg of Trypsin/Lys-C. Proteins were digested for 4 h at 37°C, 300 rpm. Then 2 μg of Trypsin/Lys-C was added again and the samples were incubated overnight at 37°C, 300 rpm. The next day, supernatants were collected after incubation on the magnetic holder. The beads were washed with 100 μl of LC–MS grade water and pooled with the supernatants. Digestion was stopped by the addition of formic acid at a final concentration of 2%. Samples were transferred back to a new tube after incubation on the magnetic stand to remove any residual beads, before being evaporated to dryness and resuspended in 10 μl of H_2_O/ACN/FA (98/2/0.1).

Peptides were analyzed using a nanoAcquity (Waters) coupled to a Q Exactive HF-X (ThermoFisher Scientific) in the same condition as upper described BioID samples.

Protein identifications were performed using Mascot (v.2.6.2). The database used contains all UniProtKB-SwissProt *Homo sapiens* entries, common MS contaminants and decoys (November 2019). A maximum of one missed cleavage was allowed, the precursor tolerance was set at 5 ppm and the fragment tolerance at 0.05 Da. Carbamidomethylation of cysteine residues was set as a fixed modification. N-term acetylation and oxidation of methionine residues were defined as variable modifications. Protein identifications were validated and relative label-free quantification was performed with Proline (v2.0; (60)) using only specific peptides without modifications and applying a 1% FDR at protein and PSM levels. Differential analysis was performed in Prostar (v1.22.6). Filtering was set to at least four values in one condition. A VSN normalisation between conditions and a quantile imputation (Quantile 2.5, Factor 1) were applied. The hypothesis test was performed using a Limma test to compare both conditions. The *P*-value was calibrated using the Benjamini-Hochberg calibration and *P*-value filtering was applied to achieve an FDR of 1.36% for a *P*-value = 1E-04. The complete dataset was deposited to the ProteomeXchange Consortium via the PRIDE partner repository with the dataset identifier PXD038967 (58,61).

### Heterologous expression and purification of mammalian proteins

To obtain the human THUMPD3-TRMT112 complex, *E. coli* BL21 (DE3) Gold cells (Agilent technologies) were co-transformed with plasmids for the expression of His_6_-ZZ tagged THUMPD3 (pMG889) and untagged TRMT112 (pFF6) (Supplementary Table S1). Protein expression was performed at 37°C in 1 l of terrific broth auto-inducible (TBAI, ForMedium; #AIMTB0260) media supplemented with kanamycin (100 μg/ml) and chloramphenicol (25 μg/ml). Bacteria were collected by centrifugation (3300 × g, 4°C, 20 min) and resuspended in lysis buffer (100 mM HEPES–NaOH pH 7.5, 200 mM NaCl; 5 mM 2-mercaptoethanol). Cell lysis was performed by sonication and cell debris were removed by centrifugation (20 000 × g, 4°C, 30 min). Soluble proteins were incubated with NiNTA agarose beads (pre-equilibrated with lysis buffer; Macherey-Nagel) for 1 h at 7°C. Washing steps, first with lysis buffer supplemented with 20 mM imidazole pH 7 and then with wash buffer (100 mM HEPES–NaOH pH 7.5, 1 M NaCl; 5 mM 2-mercaptoethanol) were used to remove non-specific proteins and nucleic acids bound to the proteins of interest. The retained proteins were eluted with lysis buffer supplemented with 400 mM imidazole pH 7 and incubated overnight at 4°C with human rhinovirus 3C protease to remove the His_6_-ZZ tag under dialysis conditions against lysis buffer. After a second incubation with NiNTA beads, the unbound proteins were collected, concentrated and diluted into buffer A (100 mM HEPES–NaOH pH 7.5, 50 mM NaCl; 5 mM 2-mercaptoethanol) before injection onto a Hitrap-Q column for ion-exchange chromatography purification. The proteins were eluted in the lysis buffer using a gradient of NaCl from 50 mM to 1 M NaCl. Finally, purification was performed on a S200-16/60 size-exclusion column (GE Healthcare) equilibrated in lysis buffer. Eluted proteins were concentrated and used for *in vitro* functional assays.

To obtain the *Bos taurus* (*Bt*) THUMPD2–TRMT112 complex, a plasmid for the co-expression of His_6_-ZZ tagged *Bt*THUMPD2 and untagged *Bt*TRMT112 was utilized (pMG1032; Supplementary Table S1). The *Bt*THUMPD2–TRMT112 complex was co-expressed in *E. coli* BL21 (DE3) Gold cells growing at first 37°C for 3 h and then transferred to 18°C for overnight culture in TBAI media containing kanamycin (100 μg/ml). For purification of the complex, the same procedure as for the human THUMPD3-TRMT112 complex was applied with the difference that the composition of the lysis buffer was 20 mM Tris–HCl pH 8, 200 mM NaCl, 5 mM 2-mercaptoethanol, 10 μM ZnCl_2_.

A pnEA-vH-based plasmid for the expression of C-terminally His_6_ tagged TRMT11 (pMG715) and the pFF6 plasmid for expression of untagged TRMT112 were co-transformed in *E. coli* BL21 (DE3) Gold cells for large-scale cell culture (1 l). Bacteria were first incubated at 37°C for 3 h and then shifted to 18°C for overnight incubation in TBAI media containing ampicillin (100 μg/ml) and chloramphenicol (25 μg/ml). The human TRMT11-TRMT112 complex was purified through affinity chromatography using NiNTA resin, ion-exchange chromatography using a heparin column and then size exclusion chromatography (S200-16/60 size-exclusion column) as described above. In this case, the lysis buffer composition was 20 mM Tris–HCl pH 7.5, 200 mM NaCl, 5 mM 2-mercaptoethanol.

### Size exclusion chromatography-multi-angle laser light scattering (SEC-MALLS)

The molecular weight of the different complexes in solution were determined by SEC-MALLS as previously described (44). Briefly, for each complex, a 100 μl sample of complex (1 mg/ml) was injected on a Superdex™ 200 Increase 10/300 GL column (GE-Healthcare) at a flow rate of 0.75 ml/min. As running buffers, we used the buffer used for the size-exclusion chromatography step performed on each complex (see above). Elution was followed by a UV–visible spectrophotometer, a RID-20A refractive index detector (Shimadzu), a MiniDawn TREOS detector (Wyatt Technology). The data were collected and processed with the program ASTRA 6.1 (Wyatt Technology). *M*_w_ was directly calculated from the absolute light scattering measurements using a d*n*/d*c* value of 0.183.

### Radioactivity-based enzymatic assay

The *in vitro* tRNA methylation assays were performed as previously described (44). Briefly, The enzymatic assays were performed by mixing 75 pmol of *E. coli* tRNA_i_^Met^ (purified as previously described; (62)) with 3 pmol of enzyme and 2 μM of SAM ((Sigma-Aldrich, #7007) including 0.2 μM of [^3^H]-SAM, Perkin Elmer) in MTase buffer (25 mM K-phosphate pH 7.5, 50 μM EDTA, 5 mM MgCl_2_, 5 mM NH_4_Cl) in a total volume of 50 μl. The reaction was incubated overnight at 37°C and stopped by adding 5 ml of cold trichloroacetic acid (TCA 5%) containing 0.5% of methionine, then 20 μl of RNA carrier (4 mg/ml, Colnbrook-Bucks-England), and followed by filtration on glass microfiber filters (Whatman GF/C). Beckman Coulter LS6500 scintillation counter was used to determine [^3^H] incorporation.

### Characterization of RNA modifications by MS

m^2^G nucleosides formed in tRNAs upon incubation with different enzymes were detected by High-performance liquid chromatography (HPLC)–MS using a modified version of the protocol published in (44). Briefly, 20 μg of tRNA substrate (for example, *E. coli* tRNA_i_^Met^; corresponding to 834 pmol of tRNA) were incubated overnight with 0.8 μg of TRMT112-MTase complexes (34 pmol of enzyme) supplemented with 126 pmol of SAM at 37°C. The negative control consisted of only tRNA in the same conditions. RNA digestion into nucleosides and HPLC–MS procedures were as previously reported (44).

For nucleoside quantification, the samples were analyzed by LC–high resolution MS (HRMS) instrument based on the specific elution time and *m/z* value of the respective nucleosides. The experiments were performed with a LC 1260 Prime (Agilent Technologies, Waldbronn, Germany) coupled to a high-resolution tandem mass spectrometer QTOF 6546 (Agilent Technologies, Waldbronn, Germany). The column (HSS T3, 150 mmm – 2.1 mm × 2.7 μm, Waters, USA) was chosen for an efficient separation of the three nucleosides without the use of any buffer such as ammonium acetate in the mobile phase. The elution gradient started from 100% water + 0.1% formic acid to 50% acetonitrile in 8 min then 100% acetonitrile during 2 min and finally back to the initial conditions. The injected volume was fixed at 2 μl. For electrospray (ESI) analysis, mass spectra were recorded in positive ion mode with the following parameters: gas temperature 325°C, drying gas flow rate 10 l min^–1^, nebulizer pressure 30 psi, sheath gas temperature 400°C, sheath gas flow rate 10 l min^–1^, capillary voltage 3500 V, nozzle voltage 500 V, fragmentor voltage 110 V, skimmer voltage 45 V, Octopole 1 RF Voltage 750 V. For ESI, internal calibration was achieved with two calibrants purine and hexakis (1 h,1 h,3 h-tetrafluoropropoxy) phosphazene (*m/z* 121.0509 and *m/z* 922.0098) providing a high mass accuracy better than 3 ppm. First of all, standards, including G (Sigma-Aldrich; #G6752), m^2^G (Sigma-Aldrich; #M4004) and m^2,2^G (Berry and associates; #PR-3702) used in this experiment, were analyzed by LC–HRMS to ensure the elution time and calibration curves from 22 to 66 000 fmol injected in triplicate were plotted for each nucleoside. For quantification, the nucleoside samples were diluted into different concentrations (as determined by the OD at 254 nm) to enable quantification on either G or m^2^G/m^2,2^G based on their relative concentrations.

The m^2^G modifications were mapped on *E. coli* tRNA_i_^Met^ by matrix-assisted laser desorption ionisation time-of-flight (MALDI-TOF) MS–MS as previously described (44).

### Knock-out HCT116 cell lines generation by CRISPR-cas9 technique

The protocol used was adapted from Ran *et al.* (63). HCT116 parental cell lines and the generated knock-out cell lines were cultured in McCoy 5A complete medium (McCoy 5A medium (Gibco) supplemented with 10% fetal bovine serum (FBS, Gibco) and 100 U/ml Penicillin and 100 μg/ml Streptomycin). The sgRNAs were designed using the CHOPCHOP website (https://chopchop.cbu.uib.no/; (64)). For each target gene, three sgRNAs selected from the top of the proposed list were cloned into the pSpCas9(BB)-2A-puro vector (Addgene plasmid #62988; (63); Supplementary Table S1).

To generate knock-out cell lines, 0.5 × 10^6^ HCT116 parental cells were seeded on 6-well plates and after 24 h incubation, transfected with 500 ng of plasmids for expression of the different sgRNAs. After 24 h, transfected cells were selected with 1.5 μg/ml of puromycin for a further 72 h. Clonal cell lines were generated either by diluting into 0.8 cells per 100 μl and seeding into a 96-well plate, or by inoculating specific numbers of cells (for example, 100, 200 and 400 cells) into 15 cm dishes. Cells were cultured for 2–3 weeks until single colonies were visible. The single colonies were transferred into 12-well plates and cultured to 80% confluence. Cells were then collected and lysed to detect the specific protein expression by western blotting. Colonies showing no expression of the protein of interest were selected for genomic DNA extraction using the DNeasy® blood and tissue kit (Qiagen) according to the manufacturer's instruction. The targeted genome regions were amplified by PCR using primers designed according to the CHOPCHOP website (Supplementary Table S2) and products were subjected to Sanger sequencing (Eurofins-MWG).

### Cell growth curve

5000 cells were plated in 12-well plates for each experiment. Cell numbers were manually counted at different time points using a hemocytometer (KOVA Glasstic Slide 10 with quantitative grid) according to the manufacturer's instructions. Briefly, cells were collected following trypsin treatment and diluted to a suitable concentration (less than 50 cells in each grid after staining). Cells were stained by adding equal volume of 0.4% Trypan blue (Gibco) and the mixture was incubated at room temperature for 5 min. The sample was added to each grid and living cells were counted manually under a microscope. The total number of cells was determined using the following formula provided by the manufacturer: Total cell numbers = the average counting cells × 90 (factor) × dilution times × 1000 (volume, μl)/81 (numbers of small grids).

### Colony formation assay

1000 cells were seeded in 6-well plates complemented with 3 ml medium. The cells were grown for around 10 days until the colonies became visible and reached a suitable size. The colonies were washed twice with PBS, fixed with 4% paraformaldehyde (PFA) for 15 min at room temperature, and stained with crystal violet solution for 10–30 min. The staining solution was then removed, and the colonies were washed gently with running water. The plate was dried at room temperature overnight and pictures were taken using a ChemiDoc (Bio-Rad).

### RNA extraction from human cell lines and enrichment of specific RNA populations

Total RNAs were extracted from the different human cell lines (grown in one dish of 10 cm or 15 cm plate) using TRIzol^TM^ LS reagent (Invitrogen) according to manufacturer's instructions. The total RNA pellet was resuspended with 20–50 μl of sterile or RNase-free water.

For small RNAs extraction, 0.1 volume of 5 M NaCl and 2 volumes of 100% cold ethanol were added to the total RNAs to precipitate the small RNAs. The RNA pellet was washed with 90% cold ethanol and then resuspended in 1 ml of 1 M NaCl solution followed by centrifugation at 20 000 × g and 4°C for 30 min to remove large RNAs. The supernatant containing small RNAs was then precipitated by 2 volumes of 100% cold ethanol followed by dissolution into 1 ml of 1.8 M Tris–HCl pH 8. The tRNAs present in these small RNAs were deacylated by incubation at 37°C for 1.5 h and precipitated by adding 0.1 ml of 5 M NaCl and 2.2 ml of 100% cold ethanol prior to storage at –20°C. The small RNAs were harvested by centrifugation at 20 000 × g and 4°C for 30 min and washed with 70% of cold ethanol. Finally, the RNA pellet was dried and dissolved in sterilized water. The composition of different RNA fractions was analyzed by a denaturing (urea) polyacrylamide gel electrophoresis (PAGE).

For total tRNA extraction, cells were lysed by incubation with buffer A (20 mM Tris–HCl pH 7.5, 10 mM KCl, 1.5 mM MgCl_2_, 1 mM DTT, 0.1% NP40) complemented with 1 × PIC at 4°C for at least 10 min. The soluble cell extract was centrifuged at 20 000 × g and 4°C for 20 min and the supernatant transferred to a new tube. 1/9 volume of 125 mM EDTA was added and the mixture was incubated on ice for 15 min followed by addition of 1/9 volume of 5 M NaCl and incubation on ice for another 10 min. Next, the sample was transferred into a polycarbonate tube and subject to ultracentrifugation at 200 000 × g for 2 h at 4°C to precipitate the large RNAs. The small RNAs present in the supernatant were extracted using TRIzol™. The extracted small RNAs (maximum amount of 100 μg of RNAs) were purified by size-exclusion chromatography column (Acclaim SEC-300; #079723) using a 100 mM NH_4_Ac buffer using a micro-Aktä machine and a 0.5 ml/min flow rate. *E. coli* tRNA_i_^Met^ was used as standard to determine the elution volume of tRNAs. The collected fractions were analyzed by denaturing PAGE to estimate the purity of the RNAs.

### tRNA quantification

Total cellular RNAs extracted using TRIzol™ LS reagent (Invitrogen) were treated with DNase I for 30 min at 37°C before a further purification step using the RNeasy® MinElute™ cleanup kit according to the manufacturer's instructions. tRNA demethylation and reverse transcription were performed with rtStar tRNA-optimized First-Strand Synthesis Kit (Arraystar, #AS-FS-004). qPCR for tRNAs was performed using the nrStar Human tRNA PCR Array (Arraystar, #AS-NR-001–1) and Arraystar SYBR Green qPCR Master Mix(ROX+) (Arraystar; #AS-MR-006–5). The data were analyzed by the ΔCt method. The ΔCt values were calculated for each tRNA using the following formula: ΔCt(tRNAx) = Ct(tRNAx) – Ct(housekeeping genes).

### Analysis of tRNA aminoacylation

Total RNA was extracted under acidic conditions using TRI Reagent® (Sigma-Aldrich) and 10 mM NaOAc (pH 5) at 4°C (65) and stored in acidic conditions (10 mM NaOAc at pH 5 and 1 mM EDTA at pH 8). As a control, a sample of the total RNA was incubated at pH 8.9 for 60 min at 37°C to achieve complete deacylation. 5 μg of each of the RNA samples were separated in an acidic (pH 5) 10% polyacrylamide 7 M urea gel at 4°C for 20 h at 170 V. After transfer to a Hybond-N membrane (Cytiva), selected tRNAs were detected by northern blotting using probes listed in Supplementary Table S4.

### Native PAGE analysis of tRNA structure

200 ng of total RNA extracted using TRI Reagent® (Sigma-Aldrich), resuspended in 10 mM Tris pH 7.4 and 50% glycerol, supplemented with bromophenol blue, were separated on 20% non-denaturing polyacrylamide gels. RNAs were transferred to Hybond-N membranes (Cytiva) and detected by northern blotting using probes listed in Supplementary Table S4.

### Polysome profiling by sucrose gradient

10 × 10^6^ cells were grown for 24 h in 15 cm plates prior to incubation with cycloheximide (100 μg/ml final concentration; Sigma-Aldrich; #C7698) for 10 min at 37°C. Cells were washed twice with 10 ml ice cold PBS supplemented with cycloheximide (100 μg/ml final concentration). 5 ml ice cold PBS supplemented with cycloheximide (100 μg/ml final concentration) were added and the cells were harvested by scraping. Cells were pelleted by centrifugation at 300 × g for 5 min at 4°C and resuspended in 400 μl of hypotonic buffer (5 mM Tris–HCl pH 7.5, 1.5 mM KCl, 2.5 mM MgCl_2_ supplemented with 1 × PIC). After addition of cycloheximide (100 μg/ml final concentration), DTT (2 mM final concentration) and RNasin (100 U), cells were vortexed for 5 s. Next, Triton-X100 and Na-deoxycholate (0.5% final concentration each) were added and vortexing repeated. After 10 min incubation at 4°C, samples were centrifuged for 5 min at 4°C and 12 000 × g. The supernatant was transferred to a pre-chilled tube and the optical density (OD) at 260 nm was measured using a Nanodrop ND-1000 spectrophotometer (Labtech). Then, 300 μl of sample at OD_260_ of 7.5 were loaded on a 15–45% sucrose gradient (prepared with a BioComp Gradient Master 107ip in 20 mM Tris–HCl pH 7.5, 100 mM KCl, 10 mM MgCl_2_, 1mM DTT, 100 μg/ml cycloheximide). After ultracentrifugation (200 000 × g for 90 min at 4°C), the fractions were collected using a Foxy Jr fraction collector (Teledyne ISCO) and the absorbance at 254 nm was measured with a UA-6 device (Teledyne ISCO).

### AHA-click labeling assay

7 × 10^5^ cells were seeded into 6-well plates and after overnight incubation, the medium was replaced by DMEM medium lacking methionine (Gibco; #21013-024) for 1 h. 100 μM l-azidohomoalanine (AHA; Sigma Aldrich; #900892) was then added for 3 h. Cells were washed twice with cold PBS and lysed in lysis buffer (50 mM Tris–HCl pH 8.0 and 1% SDS) followed by sonication for 2 min. The soluble cell extract was separated by centrifugation at 20 000 × g and 4°C for 10 min and transferred into a new tube. Protein concentration was determined by absorbance measurements at 280 nm using a Nanodrop ND-1000 spectrophotometer (Labtech). Equal amounts of cell extracts (100 μl at 1.5 μg/μl) were prepared for all samples. 100 μM Biotin-Alkyne (Sigma-Aldrich; #764213), 1 mM TCEP (Sigma-Aldrich; #C4706), 100 μM THPTA (Sigma-Aldrich; #762342) and 1 mM CuSO_4_ (Sigma-Aldrich; #C8027) were sequentially added to the protein samples, followed by a short vortex for several seconds. The mixture was incubated at room temperature for 1 h. After that, 4 volumes of methanol were used to precipitate proteins. The samples were stored overnight at –20°C and then, the protein pellet was collected by centrifugation (20 000 × g, 4°C, 30 min) and dried at room temperature. The protein pellet was next denatured by adding 2 × loading buffer (40 μl per 100 μl mixture) followed by heating at 95°C for 5 min. The samples were separated by SDS-PAGE (12%) and analyzed by western blotting using HRP-conjugated streptavidin at 4°C for overnight. Newly-synthesized proteins were visualized by ECL incubation and exposure. After stripping, the membranes were incubated with anti-GAPDH or anti-α-tubulin for normalization (Supplementary Table S3).

### Stably transfected HEK293 Flp-in cell lines

To generate stably transfected cell lines for the tetracycline-inducible expression of N-terminally 2 × Flag-His_6_ or C-terminally His_6_-2 × Flag tagged THUMPD2, the HEK293 Flp-In T-Rex system (Invitrogen) was used. HEK293 Flp-In cells seeded 24 h prior were transfected with appropriate pcDNA5-based plasmids as well as a plasmid for expression of the Flp recombinase using X-tremeGENE 9 DNA transfection reagent (Roche) and transfected cells were selected with hygromycin B and blasticidin S according to the manufacturer's instructions. Expression of the tagged proteins was induced by treatment with 1 μg/μl tetracycline for 24 h before harvesting.

### RNA-IP after cross-linking

To detect RNAs directly bound by THUMPD2, HEK293 cells expressing THUMPD2-His_6_-2 × Flag, 2 × Flag-His_6_-THUMPD2 or the His_6_-2 × FLAG tag were irradiated with UV light at 254 nm 3× at 800 mJ/cm^2^ to covalently crosslink proteins to their associated RNAs. Cells were lysed in a buffer containing 50 mM Tris–HCl pH 7.6, 150 mM NaCl, 0.1% NP-40, 5 mM 2-mercaptoethanol and protease inhibitors (Roche) by sonication. Protein-RNA complexes were affinity purified on anti-Flag magnetic beads (Sigma-Aldrich) then eluted overnight with 3 × Flag peptide (Sigma-Aldrich). RNA-protein complexes were then immobilized on Ni-NTA (Qiagen) in denaturing conditions (6 M guanidium–HCl) before elution of RNAs using Proteinase K. RNAs were extracted from input and eluate samples using phenol-chloroform, separated by denaturing PAGE and analyzed by northern blotting using probes listed in Supplementary Table S4.

### Purification of the U6 snRNA

Parental and THUMPD2 KO1 HCT116 cells were lysed in a buffer containing 10 mM Tris–HCl pH 8.4, 140 mM NaCl, 1.5 mM MgCl_2_, 0.5 mM EDTA, 0.5 mM DTT, 0.5% NP40 on ice for 3 min. Nuclei were pelleted by centrifugation at 5900 × g for 5 min at 4°C and the supernatant discarded. The nuclear pellet was resuspended in lysis buffer supplemented with 1.5 mM CaCl_2_ and the centrifugation step was repeated. The supernatant was removed and nuclear RNA extracted from the pellet using TRI reagent (Sigma-Aldrich) according to the manufacturer's instructions. Nuclear small RNAs (<200 nt) were then enriched using the mirVANA kit (ThermoFischer Scientific) according to the manufacturer's guidelines. To specifically enrich the U6 snRNA, hydrophilic streptavidin magnetic beads (NEB) were washed in 10 mM Tris–HCl pH 7.4 and incubated with 200 μM biotinylated antisense DNA oligonucleotide (5’-Biotin-TTTTAGTATATGTGCTGCCGAAGCGAGCAC-3’) for 90 min at room temperature. The beads were washed five times with 10 mM Tris–HCl pH 7.4 and resuspended in 6 × NTE buffer (120 mM Tris–HCl pH 7.4, 1.2 M NaCl, 15 mM EDTA). The extracted small nuclear RNAs were added to the oligonucleotide-conjugated beads and incubated at 70°C for 30 min before slowly cooling to room temperature. The supernatant was removed and the beads washed sequentially once with 1 × NTE buffer, once with 3 × NTE buffer and twice with 1 × NTE. RNAs were then eluted using 0.1 × NTE buffer at 70°C for 15 min. RNAs were precipitated using ethanol and resuspended in RNase-free water. The purified U6 snRNA was digested into nucleosides as described above. For m^2^G detection by LC–HRMS, the same protocol as described above was performed.

### mRNA-seq

Total RNA was extracted from wild-type (WT) HCT116, THUMPD2 KO1 and KO2 cells using TRI reagent (Sigma-Aldrich) according to the manufacturer's instructions. A DNase digest was performed using TURBO DNase (Ambion) for 15 min at 37°C and RNAs were purified using the RNA clean and concentrator kit (Zymo) following the manufacturer's protocol. Polyadenylated RNAs were enriched and a cDNA library prepared using the TrueSeq Stranded Total RNA kit (Illumina). 50 nucleotide, unstranded, single-end sequencing was performed using the TruSeq SBS Kit v3 on a HiSeq4000. Library preparation, quality control and sequencing were performed at the NGS Integrative Genomics (NIG) Core Unit of the University Medical Center Göttingen (UMG).

### mRNA-seq mapping and expression quantification

FASTQC from the FASTX toolkit was run to ensure high quality of the data before mapping (http://hannonlab.cshl.edu/fastx_toolkit/index.html). For mapping, the aligner STAR v2.7.10a with the following settings was used: –outFilterMultimapNmax 10 –outSAMattributes All –outSAMtype BAM SortedByCoordinate –outReadsUnmapped Fastx –chimSegmentMin 20 –chimOutType WithinBAM Junctions –quantMode TranscriptomeSAM GeneCounts –outWigType bedGraph –outWigNorm RPM –outWigStrand Unstranded –outFilterMismatchNmax 2 (66,67). UCSC Genome Browser tools bedSort and bedGraphToBigWig were used to convert from bedgraph format to bigwig format for visualization of the data in the IGV genome browser (68,69). Genome assembly version GRCh38.p13 and the GENCODE gene set from release 39 were used and gene, exon, intron and transcript annotations from ensembl, entrez and hgnc were obtained with the Bioconductor package biomaRt (70,71). SnoRNA annotations were retrieved from the GENCODE annotation gtf file. Genes containing introns spliced by the minor spliceosomes were retrieved from the Minor Intron Database (MIDB; (72); Olthof *et al.*; https://doi.org/10.1101/2022.10.12.511939).

To assess the presence of the single nucleotide insertion in all replicates of THUMPD2 KO1, we used sam- and bedtools to extract all reads overlapping chr2:39761363–39761365 (66,67). The fraction of reads with an insertion and overlapping the region of interest is given in Supplementary Figure S7C. To ease analysis soft-clipped reads were excluded from the quantification in WT and KO1.

Gene expression was quantified using the DESeqDataSetFromHTSeqCount, DESeq and results functions from the DESeq2 package in Bioconductor (73). Read counts per gene were obtained during the STAR mapping step. Only genes with 50 or more reads were kept for downstream analysis and plotting. Genomic regions with an FDR <0.05 and a log_2_-fold change greater or smaller than 0.5 were used for the differential expression comparison in Figure 5A.

### Alternative splicing analysis with rMATS and FRASER

For exon-centric analysis of alternative splicing changes, rMATS.4.0.2 was used with default settings for unstranded single-end RNA-seq data and at least a median of 5 reads per splicing event across replicates was required to consider events for further analysis (Supplementary Table S10). Intron-focused alternative splicing was performed in the software package FRASER (74,75) (see Supplementary data and  Supplementary Tables S11 and S12). The significance of splice site usage determined by FRASER between the WT, THUMPD2 KO1 and KO2 were assessed using the two-tailed Student's t-Test for splice sites that had coverage of 20 or more reads in all replicates.

For the analysis of intron retention, only introns that had only one reported 5’ and 3’ splice site in our data were considered. Splice site scores were calculated with the MaxEnt software (76). Polypyrimidine tract length was computed in an interval of 50 nt upstream of 3’ splice sites. To this end, the fraction of C or U nucleotides (PY-fraction) in sliding 5 nt windows across the 50 nt interval was calculated. The distance in nt from the nucleotide position where the PY-fraction increased to 0.5 or higher and stayed high to the end of the intron was taken as PY-tract length.

The modified *Z*-score was calculated to transform intron feature data onto a uniform scale for visualization, using the following formula (77):


(1) \begin{eqnarray*}{z}_i = \frac{{{x}_i - \bar{x}}}{{MAD}}\end{eqnarray*}

with ${x}_i$ being the median of the intron feature in group i, $\bar{x}$ being the intron feature median for all introns and MAD the median of absolute deviation.


(2) \begin{eqnarray*}MAD{\mathrm{\ }} = {\mathrm{\ }}median\left( {\left| {{x}_i - m} \right|} \right)\end{eqnarray*}

with m being all intron feature values.

To test for statistical significance, we performed the Wilcoxon-rank sum test between groups that showed increased (up) or decreased (down) intron retention (based on theta3, Figure 5G, column 3 in the *P*-value heatmap)/alternative splice site usage (based on psi3, Supplementary Figure S9E, column 3 in the *P*-value heatmap). We also tested for significance between the not significantly different group of splice sites and the up or down group, respectively (columns 1 & 2 in the *P*-value heatmaps in Figure 5G and Supplementary Figure S9E).

All mRNA-seq data analysis was done using bash scripts, software as cited above and Rstudio (2022.02.0 + 443). The R packages pheatmap and ggplot2 were used for data visualization. For sashimi plot visualization, the command-line tool ggsashimi was used (78). The high-performance cluster SCC maintained by the GWDG at the University of Göttingen was used for mapping, rMATS analysis and file format conversions.

### Validation of mRNA-seq data by RT-PCR

250 ng of purified, DNase-treated total RNA reverse transcribed with 50 pmol oligo-dT primer (5’- TTTTTTTTTTTTTTTTTTTTTTTTVN-3’; V – G/A/C, N – G/A/C/T) and Superscript III reverse transcriptase according to the manufacturer's instructions. The resultant cDNA (diluted 1:2.5) was used as a template for PCR with Phusion polymerase and dedicated oligonucleotide (Supplementary Table S4). The number of cycles was titrated to ensure exponential amplification of both products for quantification and 25 cycles yielded the best results for the three tested genes. PCR products were separated by agarose gel electrophoresis and detected using SybrGold Nucleic Acid Gel Stain (Thermofisher Scientific). Intensity quantification of individual bands was done with Image Studio Lite.

## RESULTS

### Identification of the human TMRT112 interaction network by proximity labelling

**Figure 1.**
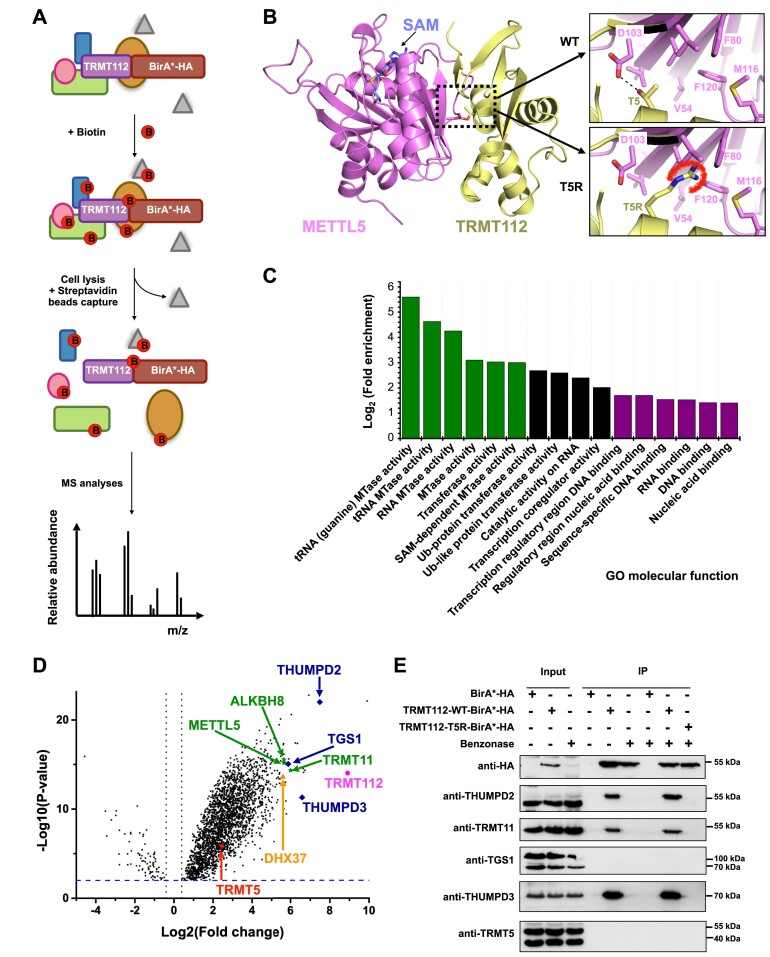
Human TRMT112 is the hub of an MTases network. (**A**) Schematic representation of the BioID-MS technique used to characterize the interaction network of human TRMT112 protein. Proteins directly or indirectly interacting with TRMT112 are depicted as rectangles and spheres, respectively. Neighboring proteins are depicted as triangles. (**B**) Representation of the 3D structure of the human METTL5-TRMT112 complex (PDB code: 6H2V; (46)). The environment of the Thr5 from TRMT112 in the complex with METTL5 is shown in the upper inset. The black dotted line represents the hydrogen bond formed by the hydroxyl group of the side chain of Thr5 and the carboxylate group of the side chain of Asp103 from METTL5. Lower inset: Model of the T5R TRMT112 mutant highlighting the steric clashes (red curved line) likely to be generated by the Arg side chain with METTL5 amino acids. (**C**) Analysis of the ‘Molecular function’ Gene Ontology (GO) terms of the 100 most enriched proteins identified in our BioID experiment. GO analysis was performed using the Panther classification system as implemented in the Gene Ontology resource (http://geneontology.org/; (116–118). Statistical analysis was performed using the Fisher's exact test and the false discovery rate (FDR). Only GO terms with fold enrichments greater than 2.5 (log_2_ value higher than 1.4) are shown. MTases and nucleic acid binding terms are colored green and purple, respectively. (**D**) Volcano plot of proteins differentially purified by TRMT112-WT-BirA*-HA *vs* controls (TRMT112-T5R-BirA*-HA, eGFP-BirA*-HA and BirA*-HA; adjusted *P*-value < 0.05). TRMT112 is shown in pink. Previously known partners of human TRMT112 protein (based on experimental evidence or similarities with yeast proteins) are indicated in green. Other highly enriched MTases are shown in blue. TRMT5, a SAM-dependent class I MTase not found among the most enriched proteins, is shown in red. The blue dotted line indicates a –log_10_(*P*-value) of 2. The black dotted lines correspond to fold changes higher than –1.32 or 1.32. Statistics were calculated from five biologically independent experiments. (**E**) Co-immunoprecipitation experiments confirming that TRMT11, THUMPD2 and THUMPD3 interact with TRMT112. The TRMT112-WT-BirA*-HA, TRMT112-T5R-BirA*-HA and BirA*-HA (control) constructs were expressed in HEK293T cells in the absence of biotin and purified using anti-HA beads. Co-immunoprecipitated proteins were detected by western blot using the indicated antibodies. Co-IP experiments were performed in the absence or presence of benzonase to exclude a role of nucleic acids in these interactions. Three biologically independent experiments were performed and a representative image is shown.

Recently, an immunoprecipitation approach coupled to stable isotope labelling by amino acids in cell culture (SILAC) was used to probe the MTase interactome of human TRMT112 (51). It is possible that MTase interaction partners with low expression levels and other transient or weak interactors are not captured by such affinity-based techniques. Therefore, to identify proteins interacting with human TRMT112 in an unbiased way in intact cells, we used the BioID (proximity-dependent biotin identification) approach coupled with mass spectrometry (MS) (Figure 1A; (79)). As the crystal structures of the human TRMT112-METTL5 (46) and TRMT112-HEMK2 (30) complexes show that the N-terminus of TRMT112 is directly engaged in the interaction with its MTase partners, the BirA* domain endowed with biotin ligase activity was fused to the C-terminus of WT TRMT112 to generate the TRMT112-WT-BirA*-HA construct. Importantly, BirA*-HA alone and eGFP-BirA*-HA were included as negative controls to eliminate background in the list of identified proteins. In addition, a TRMT112-T5R-BirA*-HA construct, designed based on the direct involvement of threonine 5 side chain in interaction with HEMK2 or METTL5 (Figure 1B), was included. As all available crystal structures of TRMT112-MTase complexes show that the MTases interact in a very similar manner with TRMT112 (17,20), the substitution of T5 by the longer and bulkier arginine side chain (T5R) was anticipated to generate steric hindrance and prevent TRMT112 interaction with MTase partners. The effectiveness of this amino acid substitution in preventing association of TRMT112 with METTL5 was confirmed by co-IP (Supplementary Figure S1A), confirming that the T5R mutant can serve as an ideal negative control for the identification of TRMT112-associated proteins.

Human HEK293T cells were transfected with appropriate amounts of each of the plasmids expressing either BirA*-HA, eGFP-BirA*-HA, TRMT112-WT-BirA*-HA or the mutant TRMT112-T5R-BirA*-HA to transiently express these four protein constructs at approximately the same level (Supplementary Figure S1B). Each condition was performed in five replicates to allow robust statistical analyses, and proteins enriched by an affinity purification step using streptavidin beads were identified by MS. A total of 2281 proteins (Fold change (FC) > 2 and *P*-value < 0.001) were specifically enriched with the TRMT112-WT-BirA*-HA protein compared to the three control conditions (Supplementary Table S5). Gene ontology ‘molecular functions’ analysis for the 100 proteins with the highest FCs revealed strong enrichment of proteins annotated as SAM-dependent MTases, in particular RNA MTases as well as proteins involved in nucleic acid binding (Figure 1C). This is in line with the known function of TRMT112 as a central activator of RNA-modifying MTases. Six MTases were identified among the 100 most enriched proteins, including ALKBH8 and METTL5 that are established TRMT112 partners (Figure 1D; (36,37,46)). TRMT11, the orthologue of yeast and archaeal Trm112-interacting Trm11 proteins, was identified, suggesting a conserved interaction (21,31,40,44). THUMPD3, a recently identified tRNA m^2^G MTase and an uncharacterized putative MTase, THUMPD2, were also recovered. Interestingly, TGS1 (Trimethyl guanosine synthetase 1), an MTase modifying the 5’ end cap of short non-coding RNAs involved in pre-mRNA splicing (U snRNAs) or ribosome biogenesis (snoRNAs; (80)) was also found to be enriched. Notably, two well-established TRMT112 MTase partners (HEMK2 and BUD23) were not identified by this BioID experiment (30,81). However, DHX37, a DEAH helicase involved in the release of the U3 snoRNP from pre-ribosomal particles and that is orthologous to yeast Dhr1, a physical partner of the Bud23-Trm112 complex (23,82–84), was found among the most enriched proteins suggesting that BUD23 and TRMT112 likely interact in these conditions but that BUD23 is resistant to biotinylation for some yet unknown reasons.

To confirm these results, TRMT112-WT-BirA*-HA or TRMT112-T5R-BirA*-HA were expressed in human cells in the absence of biotin and complexes immunoprecipitated via the TRMT112 HA tag in the presence or absence of the benzonase nuclease. The presence of the MTases identified by proximity labelling in the immunoprecipitated fraction was determined by western blotting (Figure 1E). With the exception of TGS1 as well as TRMT5, an MTase present in the enriched proteins but not among the most enriched, all the MTases tested (TRMT11, THUMPD3 and THUMPD2) co-precipitated with WT TRMT112 but not with the T5R mutant, indicating a specific interaction. These interactions were not affected by nuclease treatment indicating that they are not bridged by nucleic acids (Figure 1E). In parallel, we performed a reverse co-IP experiment using 3 × Flag-THUMPD2 or -THUMPD3 as bait and detected TRMT112 in the IP fractions, confirming the interaction of TRMT112 with THUMPD2 and THUMPD3 (Supplementary Figure S1C).

Consistent with a study published during the preparation of this article (51), these results suggest that the MTase interactome of TRMT112 comprises at least seven RNA MTases, including the recently characterized tRNA MTase THUMPD3 (52) and the currently uncharacterized THUMPD2. Our proximity labelling data are also a useful resource for learning more about the functions and cellular context of TRMT112-MTase complexes and their regulation (see Discussion).

### The TRMT11-TRMT112 and THUMPD3-TRMT112 complexes are m^2^G tRNA MTases

**Figure 2.**
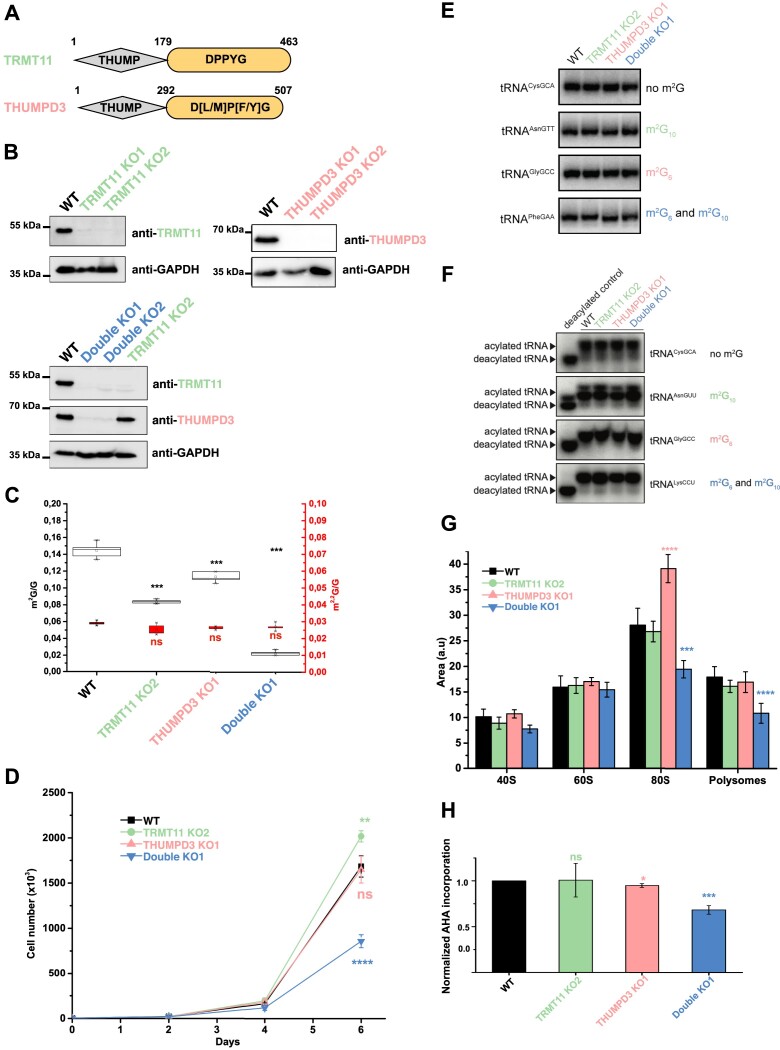
tRNA m^2^G modifications are important for proliferation of HCT116 colorectal cancer cells and optimal translation. (**A**) Schematic representation of TRMT11 and THUMPD3 showing their domain organization. The predicted amino acid boundaries of the different domains are shown above each diagram. The MTase domains are colored in orange. The strongly conserved signature found in the MTase domains, which is assumed to coordinate the substrate into the MTase active site, is indicated. (**B**) Western blot analyses of HCT116 TRMT11 (top left panel), THUMPD3 (top right panel) or double (lower panel) knockout cell lines. Proteins of interest were detected by western blot using the indicated antibodies. Three biologically independent experiments were performed and a representative image is shown. (**C**) Quantification by LC–HRMS of m^2^G (left black axis) and m^2,2^G (right red axis) levels in total tRNAs purified from the indicated cell lines. Mean values calculated from five replicates are shown and error bars represent standard deviation. (**D**) Growth analysis of WT, TRMT11, THUMPD3 or TRMT11/THUMPD3 KO HCT116 cell lines. Cell numbers were monitored every 24 h for 6 days. Three biologically independent experiments were performed and error bars represent standard deviations. (**E**) Total RNAs extracted from the indicated cell lines were separated by native polyacrylamide gel electrophoresis and analyzed by northern blotting using probes hybridizing to the tRNAs indicated on the left. The presence and position of m^2^G in the detected tRNAs is noted on the right. Three biologically independent experiments were performed and a representative image is shown. (**F**) Total RNAs were extracted from the indicated cell lines under acidic conditions. A sample subjected to alkaline treatment served as a deacylated control. Samples were separated by denaturing polyacrylamide gel electrophoresis under acidic conditions before analysis by northern blotting using probes hybridizing to the tRNAs indicated on the right. The presence and position of m^2^G in the detected tRNAs is also given. Three biologically independent experiments were performed and a representative image is shown. (**G**) Polysome profile analyses using the indicated cell lines were performed. For each cell line, the mean areas of each of the ribosomal complexes (small subunit – 40S, large subunit – 60S, monosomes – 80S and polysomes) observed in the polysome profile analyses for each cell line is shown. Non-statistically significant differences are not indicated for the sake of clarity. Five biologically independent experiments were performed and error bars represent standard deviations. (**H**) Effect of TRMT11 and/or THUMPD3 depletion on overall protein synthesis. For TRMT11 KO2 and TRMT11/THUMPD3 KO1 cell lines, the GAPDH signal was used to normalize the data, whereas for the THUMPD3 KO1 cell line, we used the alpha-tubulin signal as GAPDH levels are affected in this cell line. Three biologically independent experiments were performed and error bars represent standard deviations.

TRMT11 and THUMPD3 both contain an N-terminal THUMP domain and a C-terminal SAM-dependent MTase domain (Figure 2A; (42,44,85)). In tRNA modifying enzymes, the THUMP domain is predicted to bind to the tRNA ‘CCA’ tail via a large positively charged region (86), while the catalytic domain modifies the base of a nucleotide located in the tRNA amino acyl acceptor stem loop. The MTase domains of these enzymes contain a highly conserved signature (Figure 2A) characteristic of enzymes methylating planar amino groups on nucleic acids, such as m^2^G (87,88). Based on its high similarity to *S. cerevisiae* Trm11 (34% and 54% sequence identity and similarity, respectively), human TRMT11 is anticipated to be responsible for m^2^G formation at position 10, but this has not yet been demonstrated (89). During the course of this study, THUMPD3, which has no yeast orthologue, was identified as the enzyme installing m^2^G at positions 6/7 of many metazoan tRNAs (52).

To analyze the functions of these two complexes, we co-expressed TRMT112 with TRMT11 or THUMPD3 and purified the stable heterodimeric complexes (Supplementary Figure S2; Supplementary Table S6). In agreement with Yang and colleagues (52), we confirmed that the THUMPD3-TRMT112 complex indeed acts as an m^2^G tRNA MTase targeting position 6 of an *E. coli* tRNA_i_^Met^ substrate using a combination of *in vitro* biochemical assays and mass spectrometry approaches (LC/MS and MALDI-MS/MS; see Supplementary text for details about the MALDI-MS/MS mapping of m^2^G on tRNA; Supplementary Figures S3A–D and S4A–C). Using the same approaches, we have characterized for the first time the enzymatic activity of the human TRMT11–TRMT112 complex. Similarly to its yeast and archaeal orthologues (31,40,44), LC/MS and MALDI-MS/MS data indicate that this complex catalyzes the formation of m^2^G at position 10 of tRNAs (Supplementary Figures S3A–D and S4D–F and Supplementary text).

To further explore the molecular functions of these m^2^G tRNA MTases in human cells, we generated HCT116 cell lines lacking TRMT11 or THUMPD3 using the CRISPR-Cas9 gene knockout technique. For each cell line, two different cell populations in which TRMT11 or THUMPD3 could not be detected in the total cell extract by western blot were obtained (KO1 and KO2; Figure 2B). In the TRMT11 KO2 cell line, the *THUMPD3* gene was further edited to create the TRMT11/THUMPD3 double KO1 and KO2 cell lines (Figure 2B). Sequencing of the targeted loci in these cell lines revealed single nucleotide insertions in these genes, resulting in translation frameshifts (Supplementary Figure S5A–C and Supplementary text for more details). We then selected one of the each of the KO cell lines, purified total tRNAs, and quantified the levels of m^2^G and m^2,2^G by LC–HRMS after digestion into nucleosides (Figure 2C). Compared to parental cell lines, lack of TRMT11 or THUMPD3 resulted in ∼40% and ∼20% decreases in the m^2^G level in total tRNAs, respectively. However, concomitant inactivation of TRMT11 and THUMPD3 resulted in an 85% decrease in m^2^G levels. These data thus confirm that human TRMT11 and THUMPD3 are the major m^2^G tRNA MTases. The residual m^2^G observed in the TRMT11/THUMPD3 double KO1 cell line may be explained by the presence of m^2^G_26_ in tRNA^ValCAC/MetCAU^, m^2^G in mitochondrial tRNAs and/or intermediates generated during the formation of m^2,2^G at position 26 of some tRNAs by TRMT1. Notably, m^2,2^G levels are not affected across these different HCT116 cell lines (Figure 2C). This confirms that, 1) TRMT11 and THUMPD3 do not catalyze the formation of m^2,2^G on tRNAs and 2) lack of m^2^G_6/7/10_ does not influence the formation of m^2,2^G at position 26 in tRNAs.

### The concomitant deletion of both m^2^G tRNA MTases affects cell proliferation and protein synthesis without significantly influencing tRNA stability, folding or aminoacylation

The molecular functions of m^2^G modifications in tRNAs remain unknown. Comparing the growth rate of the TRMT11, THUMPD3 and double KO cell lines demonstrated that lack of THUMPD3 or TRTM11 did not, or only mildly, affected the growth of HCT116 cells, respectively (Figure 2D). However, lack of both TRMT11 and THUMPD3 strongly reduced cell proliferation, revealing that the concomitant presence of m^2^G modifications at tRNA positions 6, 7 and 10 is biologically important. Consistent with this, the double KO cell line formed markedly smaller colonies on soft agar, confirming the profound proliferation defects of this cell line devoid of m^2^G modifications in the acceptor stem/D-arm (Supplementary Figure S5D).

As presence of modified nucleotides can influence tRNA stability or folding (90,91), tRNA levels in the KO cell lines were first comprehensively determined using the nrStar™ human/mouse tRNA PCR array service from Arraystar Inc. (Rockville, MD 20850, USA; Supplementary Figure S6A–C and Supplementary Table S7). No significant alterations in the levels of tRNAs lacking m^2^G_6/7/10_ modifications were observed, indicating that they are not targeted for degradation. tRNA modifications can also influence folding (92) so RNAs extracted from the WT and KO cell lines were separated by native polyacrylamide gel electrophoresis under conditions that allow modification-dependent alterations in tRNA structure to be detected (Supplementary Figure S6D; (92)) and selected tRNAs detected by northern blotting. However, the migration patterns of m^2^G_6_-, m^2^G_10_-, m^2^G_6+10_- and non-m^2^G-containing tRNAs did not differ, suggesting that m^2^G modifications do not strongly influence the structure of these tRNAs (Figure 2E). To fulfill their role as adaptors between mRNAs and nascent peptides in the context of translation, tRNAs are aminoacylated and, in some cases, tRNA modifications can influence aminoacylation (93,94). Aminoacylated tRNAs were purified from WT and KO cells by RNA extraction under acidic conditions, which do not disrupt covalently bound amino acids. RNAs were then separated by acidic polyacrylamide gel electrophoresis alongside control samples that had been deacylated by alkaline treatment. Detection of m^2^G_6_-, m^2^G_10_-, m^2^G_6+10_- and non-m^2^G-containing tRNAs (tRNA^GlyGCC^, tRNA^AsnGUU^, tRNA^LysCCU^, tRNA^CysGCA^, respectively) revealed that these tRNAs are efficiently aminoacylated in the presence and absence of m^2^G_6/10_ modifications (Figure 2F).

To explore further whether m^2^G tRNA modifications are required for protein synthesis, the effect of lack of the relevant MTases on ribosome and polysome levels was determined. As anticipated for tRNA MTases, polysome profile analyses using sucrose gradients revealed no changes in the levels of 40S and 60S subunits in any of the cell lines (Figure 2G; Supplementary Figure S6E). Although no changes in monosome (80S) or polysome levels were detected in cells lacking TRMT11, the absence of THUMPD3 caused a significant accumulation of monosomes (Figure 2G; Supplementary Figure S6E), potentially indicating stalling of translation. Strikingly, the numbers of monosomes (80S) and polysomes were reduced in the TRMT11/THUMPD3 double KO1 cell line (Figure 2G; Supplementary Figure S6E), suggesting that translation is affected in this double KO cell line. Impaired translation will manifest in reduced protein synthesis so to analyze nascent protein production in cells lacking m^2^G_6/7/10_, the incorporation of azidohomoalanine (or AHA), a methionine analogue that can be labeled by biotin using click-chemistry, into newly synthesized proteins was monitored. This confirmed that protein synthesis is significantly reduced (∼30% decrease) in the TRMT11/THUMPD3 double KO1 cell line, while it is unaffected in the TRMT11 KO2 cell line and only slightly reduced in the THUMPD3 KO1 cell line (Figure 2H).

Altogether, these data reveal that individually, lack of either the TRMT11 and THUMPD3 m^2^G tRNA MTases does not strongly affect either cell proliferation or mRNA translation. However, their concomitant absence significantly reduces both cell growth and protein synthesis, demonstrating the combined importance of these two tRNA modifications for eukaryotic cells.

### THUMPD2 is an active m^2^G MTase with a small RNA substrate other than tRNAs

**Figure 3.**
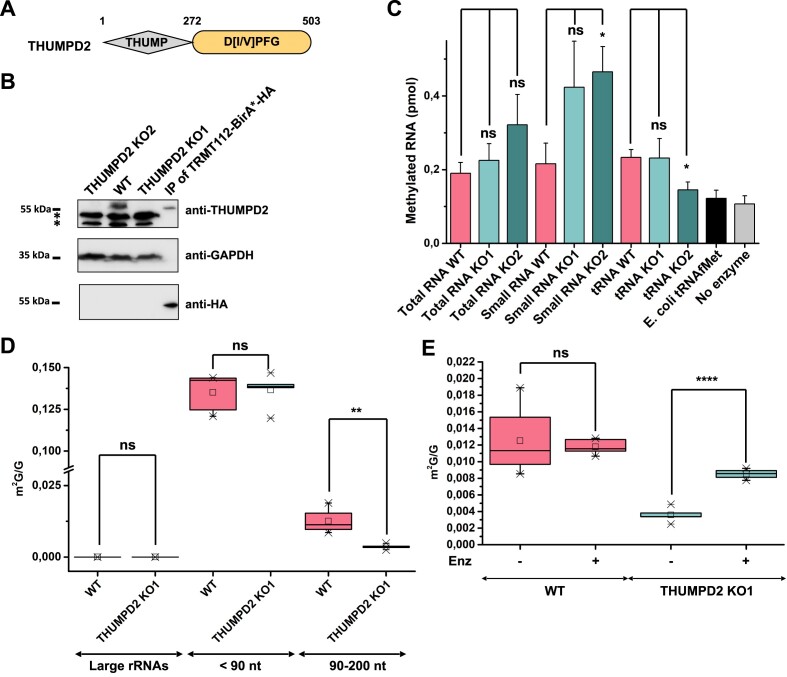
THUMPD2 is a small RNA m^2^G methyltransferase. (**A**) Schematic representation of THUMPD2 showing its domain organization. The predicted amino acid boundaries of the different domains are indicated. The MTase domain is colored in orange. The strongly conserved signature found in the MTase domain, which is assumed to coordinate the substrate into the MTase active site is indicated. (**B**) Western blot analysis of proteins from WT HCT116 and the THUMPD2 KO1 and KO2 cell lines. Proteins of interest were detected using indicated antibodies. The GAPDH signal was used as a loading control. Due to the presence of two major contaminant bands (*) detected by the anti-THUMPD2 antibody around the THUMPD2 expected molecular weight, the elution of a co-immunoprecipitation experiment performed with HA-tagged TRMT112 (right lane: IP of TRMT112-BirA*-HA) was analyzed alongside to confirm that the band absent in the THUMPD2 KO1 and KO2 samples corresponds to endogenous THUMPD2. (**C**) *In vitro* enzymatic assays performed using the recombinant bovine THUMPD2–TRMT112 complex and specific RNA populations enriched from WT HCT116 and the THUMPD2 KO1 and KO2 cell lines as substrate. Error bars represent standard deviation calculated from three biological replicates. (**D**) Quantification by LC–HRMS of m^2^G levels in different RNA species purified from the parental (WT) or THUMPD2 KO1 HCT116 cell lines. Error bars represent standard deviation calculated from five biological replicates. (**E**) Quantification by LC–HRMS of m^2^G formation upon *in vitro* incubation of small RNAs (100–150 nucleotides) purified from the parental (WT) or THUMPD2 KO1 HCT116 cell lines, with recombinant bovine THUMPD2–TRMT112 complex. Error bars represent standard deviation calculated from five biological replicates.

The remaining MTase found associated with TRMT112, THUMPD2, has so far remained uncharacterized. Similar to TRMT11 and THUMPD3, THUMPD2 contains an N-terminal THUMP domain and a C-terminal SAM-dependent MTase domain with the signature characteristic of enzymes methylating planar amino groups on nucleic acids (Figure 3A; (87)). To gain insight into its function, *THUMPD2* knockout HCT116 cell lines were generated by CRISPR-Cas9 as previously done for the tRNA m^2^G MTases. Two different cell lines in which THUMPD2 could not be detected in the total cell extract by western blot were obtained (THUMPD2 KO1 and KO2; Figure 3B). Sequencing of the targeted locus in these cell lines revealed a single nucleotide insertion resulting in a translation frameshift and premature stop codon in the case of the KO1 cell line and a three-nucleotide insertion encoding a premature stop codon in the KO2 cell line (Supplementary Figure S7A and see Supplementary text for more details).

In parallel, despite many efforts, the recombinant human THUMPD2–TRMT112 complex could not be purified because THUMPD2 was not soluble under the different experimental conditions tested, either alone or when co-expressed with TRMT112. As other protein partners may be important for THUMPD2 protein solubility, IP experiments of transiently expressed 3 × Flag-THUMPD2 protein in HEK293T cells were performed, followed by MS analysis to identify associated proteins. However, the only protein significantly enriched with THUMPD2 was TRMT112, implying that the latter protein is the only stable direct partner of THUMPD2 (Supplementary Figure S8A and Supplementary Table S8). Therefore, we purified the heterodimeric *Bos taurus* complex (*Bt*THUMPD2–TRMT112) after co-expression in *E. coli* cells (Supplementary Figure S8B and Supplementary Table S6).

To determine whether THUMPD2 is an active MTase and, if so, identify its targets, different RNA populations (total RNAs, small RNAs, or tRNAs; see Materials and Methods for preparation details) were purified from the WT and THUMPD2 KO1 or KO2 cell lines. Assuming THUMPD2 is an active MTase, RNAs from WT cells will already contain endogenously installed modifications while those from the THUMPD2 KO cell lines will lack these modifications and therefore represent potential substrates for the recombinant *Bt*THUMPD2–TRMT112 complex in *in vitro* enzymatic assays. Only minimal changes in the extent of methylation of total RNAs purified from the different cell lines were observed after *in vitro* methylation and no increases in tRNA methylation were detected in the THUMPD2 KO cell lines compared to the WT (Figure 3C). However, significant MTase activity of the recombinant *Bt*THUMPD2–TRMT112 complex was observed on the enriched population of small RNAs purified from the THUMPD2 KO2 cell line compared to the WT, and methylation of small RNAs from the THUMPD2 KO1 cell line were also notably increased (Figure 3C). This indicates that the THUMPD2–TRMT112 complex has RNA MTase activity most likely targeting small RNAs that are not tRNAs.

In parallel, small RNAs enriched from the WT and THUMPD2 KO1 cell lines were subjected to size exclusion chromatography to more precisely separate different RNA species based on their lengths: large rRNAs present as contaminants in the small RNA fraction, RNAs shorter than 90 nt (mostly tRNAs) and RNAs of intermediate size (90–200 nt; Supplementary Figure S8C, D). These different RNA pools were digested into nucleosides to quantify m^2^G levels by LC–HRMS. As expected, m^2^G was not detected in the fraction corresponding to large rRNAs (Figure 3D). Depletion of THUMPD2 also did not affect m^2^G levels in the fraction comprising RNAs shorter than 90 nt compared with the parental cell line, confirming that THUMPD2 is not an m^2^G tRNA MTase (52). However, while m^2^G was detected in the 90–200 nt long RNAs extracted from parental cell lines, it was almost undetectable in the equivalent RNA pool purified from the THUMPD2 KO1 cell line (Figure 3D). Furthermore, incubation of the 90–200 nt long RNAs purified from the THUMPD2 KO1 or parental cell lines with the recombinant *Bt*THUMPD2–TRMT112 complex resulted in a specific increase in m^2^G levels only for RNAs extracted from the THUMPD2 KO1 cell line (Figure 3E).

Taken together, these results reveal that THUMPD2 is an active m^2^G MTase with a small RNA substrate of 90–200 nt.

### THUMPD2 is the elusive m^2^G MTase targeting the U6 snRNA

**Figure 4.**
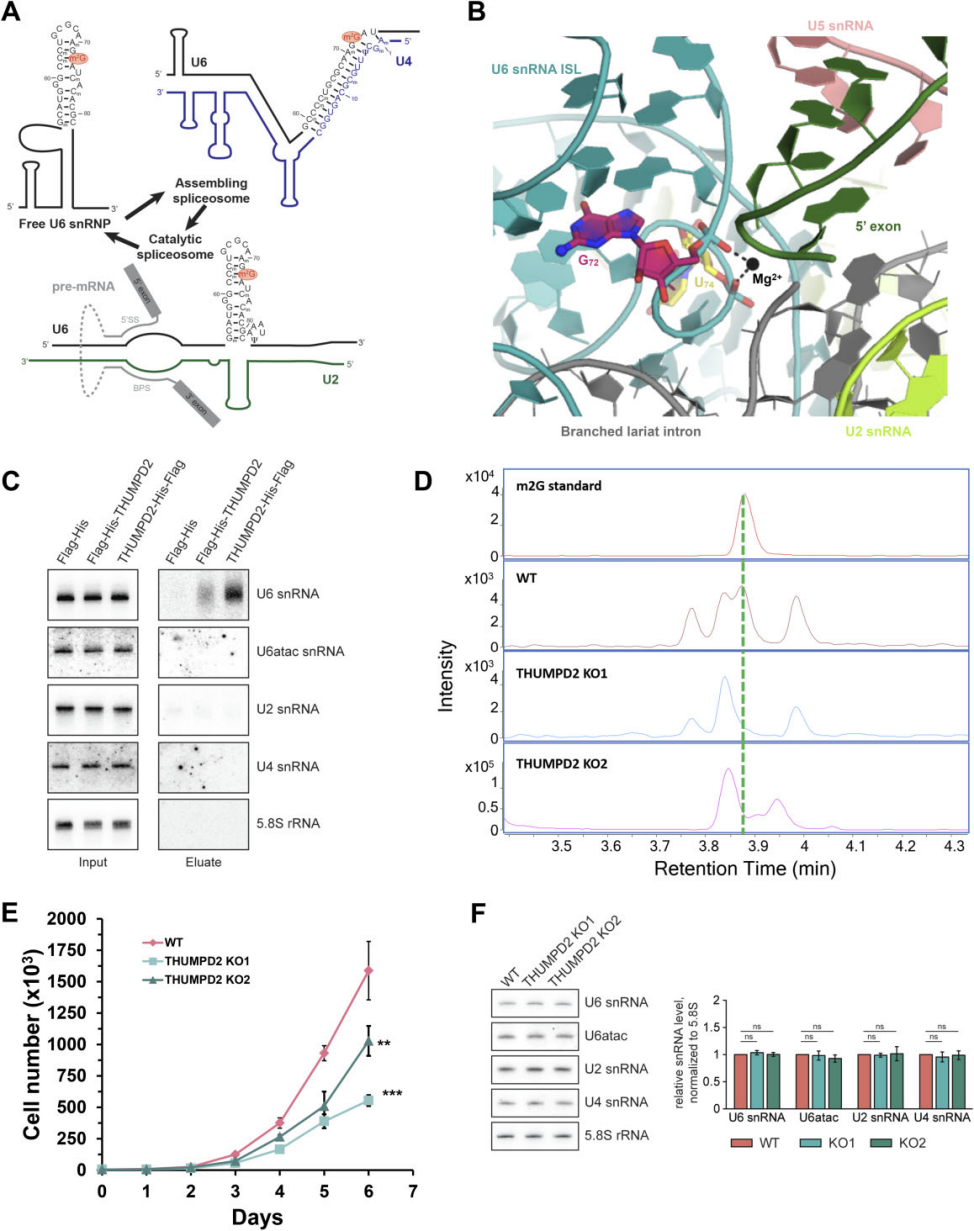
The human U6 snRNA contains an m^2^G dependent on THUMPD2. (**A**) Schematic secondary structure models of the U6 snRNA in different conformations. The position of m^2^G_72_ is highlighted in red and circled. (**B**) Cryo-EM structure of the RNA components found in the active site of the C complex of the human spliceosome (PDB code: 6ZYM; (110)). The G_72_ and U_74_ positions from U6 snRNA are shown as sticks and the catalytic metal ion (Mg^2+^) coordinated (black dashed lines) by their phosphate groups is shown as a black sphere. The N^2^ atom from G_72_, which is methylated by THUMPD2–TRMT112 complex to form m^2^G is shown as a sphere. (**C**) RNA-immunoprecipitation after UV cross-linking (254 nm) revealing a specific interaction of human THUMPD2 with U6 snRNA. Input and eluate samples were separated by denaturing polyacrylamide gel electrophoresis and the indicated RNAs were detected by northern blotting using specific oligonucleotides. Two independent experiments were performed and a representative image is shown. (**D**) LC–HRMS elution profiles of m^2^G standard (upper panel) or nucleosides obtained upon digestion of U6 snRNA purified from WT (second panel) and THUMPD2 KO1 (third panel) or THUMPD2 KO2 (bottom panel) HCT116 cell lines. The absolute intensity of *m*/*z* values of 298^+^ (corresponding to m^2^G protonated ion) is plotted. The dashed green line indicates the elution volume of m^2^G. Three independent experiments were performed and a representative image is shown. (**E**) Growth analysis of WT and THUMPD2 KO1 and KO2 HCT116 cell lines. Cell numbers were monitored every 24 h for 6 days. Error bars represent standard deviation calculated from three biological replicates. F. U6 stability is not affected by the absence of THUMPD2 protein. Northern blot showing the abundance of the U6, U6atac, U2 and U4 snRNAs and the 5.8S rRNA (loading control) in the WT and THUMPD2 KO1 and KO2 cell lines. Three independent experiments were performed and a representative image is shown. The relative, normalized level of the different snRNAs in the THUMPD2 KO1 and KO2 cell lines compared to WT is quantified. Error bars represent standard deviation calculated from three biological replicates.

The finding that THUMPD2 targets small RNAs other than tRNAs is particularly interesting because an m^2^G modification was described in 1980 in mouse and rat U6 snRNAs, which are 108 nt in size (53,54). The U6 snRNA m^2^G modification was mapped at position 72, however, the enzyme responsible for introducing this modification is still unknown (5,53,54). The U6 snRNA plays a critical role in pre-mRNA splicing by dynamically interacting with other snRNAs and protein partners to form the catalytic center of the spliceosome (95,96). In mouse, the U6 snRNA m^2^G_72_ modification is located two nucleotides upstream of U_74_, which is essential for the formation of the catalytic U6 snRNA triplex and the binding of two Mg^2+^ ions, which are directly involved in splicing catalysis (Figure 4A-B; (97)). THUMPD2 localizes to the nucleus (51) and we therefore investigated whether THUMPD2 could be responsible for m^2^G formation on the snRNA U6 by first ascertaining if this MTase physically associates with the U6 snRNA. Stably transfected cell lines expressing N-terminally 2xFlag-His_6_- or C-terminally His_6_-2xFlag-tagged versions of THUMPD2 were generated. These cell lines were subjected to UV cross-linking to covalently attach proteins to bound RNAs and extracts were then used for RNA immunoprecipitation experiments under strongly denaturing conditions to identify RNAs directly bound by this MTase. Northern blotting of input and eluate samples revealed that the U6 snRNA specifically interacts with THUMPD2 while other snRNAs and the 5.8S rRNA do not (Figure 4C). Notably, the U6atac snRNA of the minor spliceosome, which also contains a G at an equivalent position but has not been reported to contain an m^2^G, was not recovered in the RNA-IP eluate (Figure 4C), implying that THUMPD2 specifically associates with the U6 snRNA of the major spliceosome.

To determine if m^2^G is present in the human U6 snRNA and, if so, whether THUMPD2 is necessary for this methylation, the U6 snRNA was purified from WT and THUMPD2 KO1 and KO2 cell lines using a biotinylated complementary DNA oligonucleotide. The recovered RNA was digested into nucleosides, and analyzed for the presence of m^2^G by LC–HRMS. The presence of m^2^G in the human U6 snRNA was confirmed in WT cells demonstrating the evolutionary conservation of this modification from mouse/rat to human (Figure 4D). However, in the U6 snRNA extracted from the THUMPD2 KO1 and KO2 cell lines, we observed a specific loss of the m^2^G nucleoside (Figure 4D). These data demonstrate that the human THUMPD2 MTase protein is necessary for m^2^G formation on U6 snRNA, an ‘orphan’ modification identified over four decades ago.

To explore the function of THUMPD2 and the m^2^G modification for which it is required on the cellular level, growth of the THUMPD2 KO1 and KO2 cell lines was analyzed. This revealed that lack of THUMPD2 in the KO1 and KO2 cell lines significantly affects proliferation compared to the WT HCT116 cell line (Figure 4E), implying an important role for this protein and probably the modification it contributes to install. We also analyzed whether the absence of THUMPD2 and the m^2^G on the U6 snRNA, affected the stability of this snRNA or others, but we did not detect a significant differences in the amounts of U6, U6atac, U2 or U4 snRNAs in THUMPD2 KO1 or KO2 cell lines compared with the parental cell line (Figure 4F), implying that the function of this modification is not to stabilize the U6 snRNA.

### THUMPD2 is important for alternative pre-mRNA splicing

**Figure 5.**
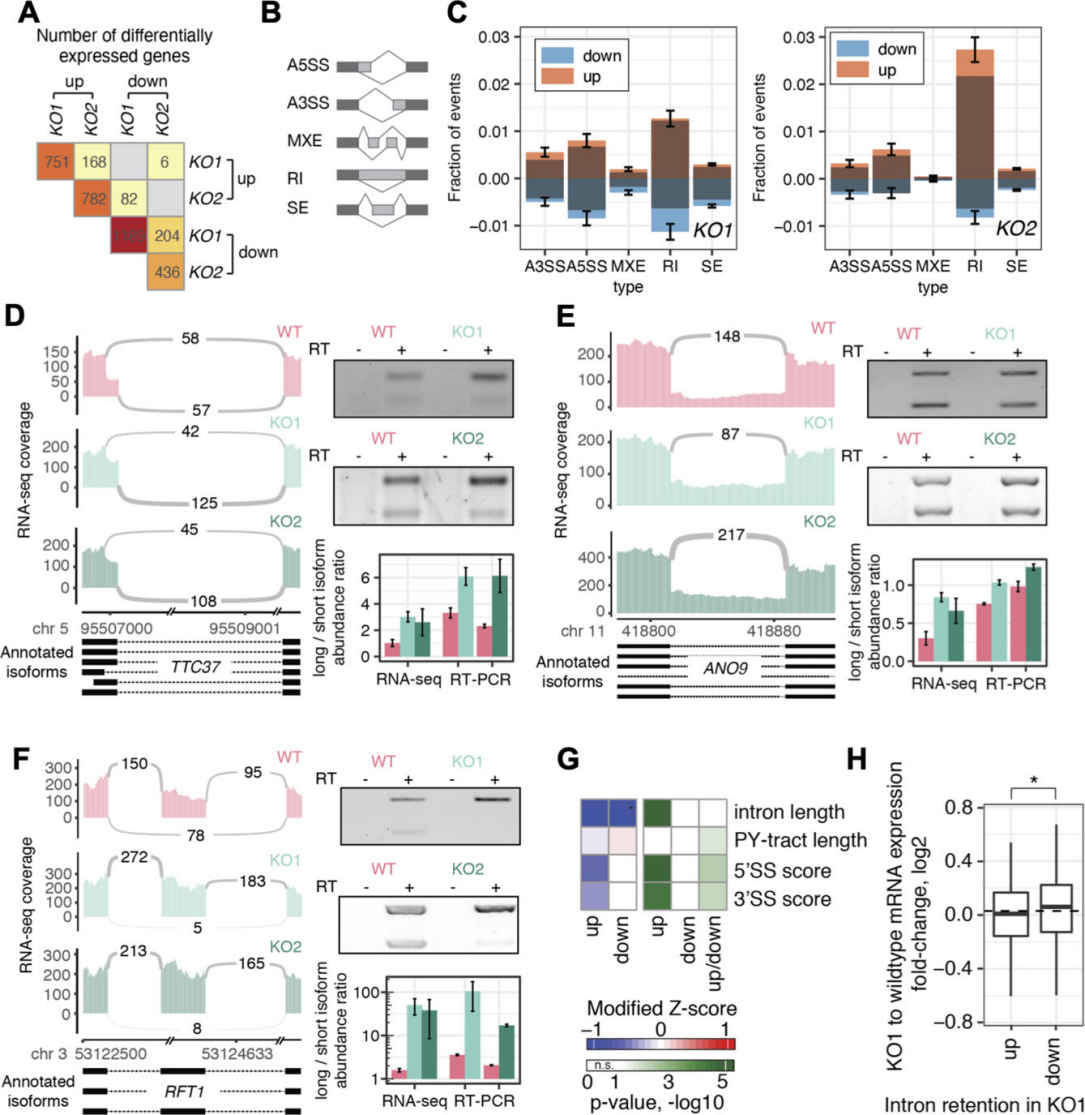
THUMPD2 is important for efficient pre-mRNA splicing. (**A**) Number of genes with significant expression changes and a log_2_-fold change of 0.5 up or down in the THUMPD2 KO1, KO2 cell lines or both. (**B**) Schematic views of different types of alternative splicing are given on the left: Alternative 5′ splice site (A5SS), alternative 3′ splice site (A3SS), mutually exclusive exon (MXE), retained intron (RI) and skipped exon (SE). (**C**) Relative fractions of significant alternative splicing events identified with rMATS for THUMPD2 KO1 (left panel) and KO2 (right panel). 95% confidence intervals are given as error bars (bootstrapping, *n* = 100). Dark bars reflect the overlap with splicing changes in the same direction for the other THUMPD2 KO cell line. (**D–F**) Visualization, validation and quantification of individual alternative splicing events by sashimi plots and RT-PCR. Sashimi plots show mRNA-seq coverage and junction counts of the pooled three replicates. Error bars reflect the standard deviation of three biological replicates. Change in A3SS in the gene TTC37 (D), RI in ANO9 (E), reduced SE/exon inclusion in RFT1 (F) in THUMPD2 KO1 and KO2 compared to WT) HCT116 cell lines. (**G**) Intron features associated with intron retention. Heatmaps show 1) the change of the median in more (up) or less (down) retained introns in THUMPD2 KO1 identified with FRASER (plotted as modified *Z*-score) relative to introns without significant changes and 2) the significance of the changes plotted as –log_10_(*P*-value) from the Wilcoxon rank-sum test. Supplementary Figure S7D shows the full distributions of all intron features without the modified *Z*-score transformation. PY – polypyrimidine, SS – splice site. (**H**) Expression changes associated with intron retention. To enhance the visibility of the global differences, outliers are not plotted here, but given in Supplementary Figure S9F. Significance was assessed with the Wilcoxon rank-sum test ((∗) *P* < 0.05). The dashed line reflects the median expression change of all other genes.

As the U6 snRNA is a core component of the catalytic spliceosome (Figure 4A, B), we explored how the lack of m^2^G in U6 affects the transcriptome. To determine mRNA levels and detect alterations in splicing in a transcriptome-wide manner, mRNA-seq analysis was performed on WT and THUMPD2 KO1 and KO2 cells. Triplicate samples were analyzed. Individual biological replicates showed good reproducibility and confirmed the genomic disruptions to the *THUMPD2* locus in the two KO cell lines (Supplementary Figure S7B–D; Supplementary Figure S9A). Global analysis of transcript levels showed several hundred transcripts with significant expression changes (Figure 5A, Supplementary Table S9). A significant portion of transcripts that showed downregulation in the THUMPD2 KO1 cell line were also downregulated in the THUMPD2 KO2 cell line, suggesting these changes represent a common phenotype arising due to lack of the m^2^G modification in the U6 snRNA (Chi-squared test, p-Value downregulated genes 4 × 10^−17^, 47% of downregulated genes in THUMPD2 KO2 common to THUMPD2 KO1). Notably, the THUMPD2 KO2 cell line displayed fewer gene expression changes than KO1 (Figure 5A). Taken together with the observation that the THUMPD2 KO1 cell line has more affected proliferation than KO2 cell line (Figure 4E), this could suggest that the THUMPD2 KO2 cell line buffers transcriptome alterations to some degree, thereby partially recovering proliferation of this cell line.

Alternative splicing, which can manifest as skipped exons, the use of alternative 5’ or 3’ splice sites, the incorporation of mutually exclusive exons or retained introns (Figure 5B), can lead to changes in transcript levels. Within the RNA-seq datasets, a number of alternative splicing events were detected as significantly different between the WT cells and those lacking THUMPD2 (KO1 and KO2; Figure 5C, Supplementary Figure S9B–C, G–H, Supplementary Tables S10 and S11). The majority of alternative splicing events significant in one of the two knock-outs changed in the same direction in the second knock-out. Intron retention was the most prevalent type of alternative splicing affected by the loss of THUMPD2 when considering the relative proportion of significantly changed events compared to all events that passed the minimal read cutoff criteria. Two different ways to quantify alternative splice site usage and intron retention supported these conclusions (rMATS and FRASER, Supplementary text, Supplementary Figures S9B–C, G–H, Supplementary Tables S10–S12).

To verify the mRNA-seq based alternative splicing analysis further, selected alternative splicing events were monitored by RT-PCR. Total RNA extracted from WT HCT116 cells and the THUMPD2 KO1 and KO2 cell lines was reverse transcribed from the poly(A) tail and the resultant cDNA served as a template for linear-range PCR with primers specific to detect the alternative 3’ splice site usage for intron 29 of *TTC37*, retention of the second to last intron of *ANO9* and skipping of exon 3 of *RTF1*. In line with the mRNA-seq data (Figure 5D–F; sashimi plots, left panels; Supplementary Table S13), this confirmed the anticipated changes in transcript isoform levels in cells lacking THUMPD2 (Figure 5D–F; right panels; Supplementary Table S13).

To gain further insights into how the absence of m^2^G in the U6 snRNAs in cells lacking THUMPD2 might influence alternative pre-mRNA splicing, key features of the introns retained in THUMPD2 KO cells were analyzed. Collectively, intron feature analysis revealed that introns retained in cells lacking THUMPD2 had lower splice site quality, shorter polypyrimidine tracts and were generally shorter than introns that did not change significantly in intron retention (Figure 5G, Supplementary Figure S9F). Various small non-coding RNAs are encoded within pre-mRNA introns and another distinguishing feature of introns retained in cells lacking THUMPD2 was the overrepresentation of those containing small nucleolar (sno)RNAs (Supplementary Figure S9I). Furthermore, gene expression was significantly reduced for genes that had two or more introns with increased intron retention compared to the group of genes with better intron removal (Figure 5H, Supplementary Figure S9F). Other feature analyses, *e.g*. for the preferential inclusion of 3’ splice sites, did not yield strong trends (Supplementary Figure S9D, E).

Altogether, these data suggest that the lack of THUMPD2-mediated m^2^G methylation of the U6 snRNA affects the constitutive splicing efficiency of introns that have suboptimal splice sites and can impact final mRNA levels.

## DISCUSSION

Around 150 different types of RNA modifications decorate transcripts from the three domains of life and additional modified nucleotides are still being identified (15,98–100). The roles of many of these modifications have been investigated first in bacteria and unicellular eukaryotes, such as the yeast *S. cerevisiae*, and are now being unraveled in metazoa, particularly in humans. The first human RNA modification enzymes to be characterized were largely orthologous to yeast proteins. However, studies performed since the early 1980s on different human and/or metazoan RNA species revealed the presence of some specific epitranscriptomic marks absent from the corresponding yeast RNAs. Consequently, the metazoan enzymes responsible for the formation of many of these ‘orphan’ RNA modifications are progressively being identified.

### TRMT112 as a common cofactor of MTases with diverse functions in regulating gene expression

**Figure 6.**
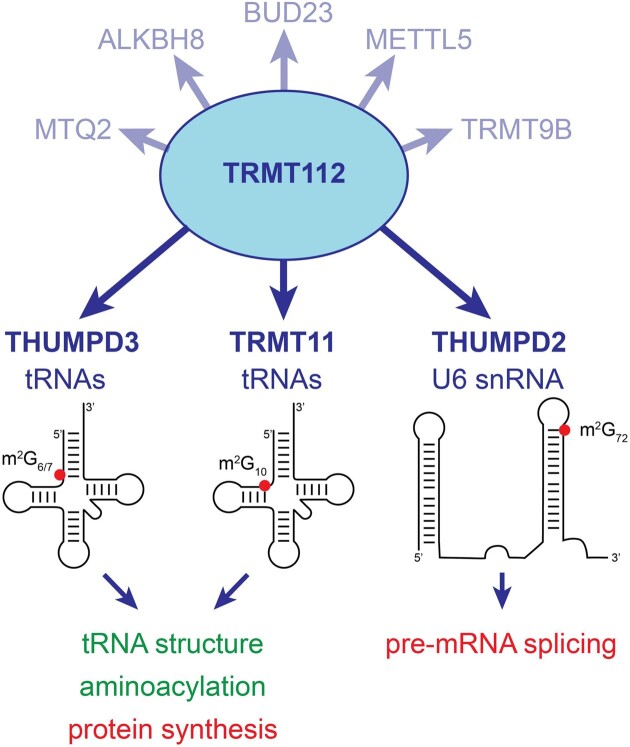
Interaction network of the human TRMT112 protein with MTases, emphasizing the m^2^G MTases. The substrates of each m^2^G MTase are indicated and a representative view of their secondary structure is shown. The locations of the m^2^G nucleotides are shown as red spheres. RNA functions affected upon depletion of the MTase are shown in red, whereas those not affected are shown in green.

In this study, we have investigated the protein interaction network of human TRMT112 (Figure 1; Supplementary Figure S1). Probing the interactome of TRMT112 in intact cells by proximity labelling, we generated a robust inventory of TRMT112-associated proteins (Supplementary Table S5), which will serve as a valuable resource to learn more about the functions and regulation of this MTase cofactor. Intriguingly, extending the list of TRMT112 interactors beyond MTases, revealed numerous post-translational modification enzymes (predominantly kinases, protein methyltransferases and components of the (de-)ubiquitination machinery) that represent potential regulators of TRMT112 and/or the various TRMT112-MTase complexes. Furthermore, numerous RNA-binding proteins linked to various different aspects of gene expression *e.g*. transcription, ribosome assembly, tRNA biogenesis, mRNA maturation, translation and RNA decay were discovered, and verification of such interactor will likely provide important insights into the context of the modification events it is involved in. The interaction network between human TRMT112 and MTases is more extensive (eight partners) than in yeast (four partners; Figure 6). Among the TRMT112 interactors identified were three additional MTases, TRMT11, THUMPD2 and THUMPD3 (Figure 1D, E), confirming recent studies (51,52). Our results indicate that these three enzymes, each composed of a class I MTase domain fused to an N-terminal THUMP domain (Figures 2A and 3A), contribute to the formation of m^2^G on RNAs (Supplementary Figure S3C-D, Figure 2C, 3C–E and 4D). We demonstrate that the TRMT112-TRMT11 and TRMT112-THUMPD3 complexes are responsible for installing m^2^G modifications in tRNAs whereas TRMT112–THUMPD2 targets the U6 snRNA (Figure 6).

These m^2^G modifications were known for several decades but analysis of their biological functions was limited by lack of knowledge of the enzymes responsible for installing them (53,54,101–103). Here, we show that these three enzymes are important for the proliferation of human HCT116 colorectal cancer cells (Figures 2D and 4E, Supplementary Figure S5D). Hence, TRMT112 emerges as an essential cofactor for methylation events on diverse non-coding RNA molecules (18S rRNA, tRNAs and snRNA U6). The discovery that TRMT112 functions as the central hub of a MTase network opens the intriguing possibility of cross-regulation of different gene expression processes via coordinated regulation of RNA modification events.

### m^2^Gs in the tRNA body function co-operatively to optimize protein synthesis for efficient cell proliferation

In this study, we functionally characterize the human TRMT11–TRMT112 complex for the first time, revealing that it catalyzes the formation of m^2^G at position 10 of tRNAs (Supplementary Figure S3B–D and Figure 2C, Supplementary Figure S4D–F), similarly to yeast and *Archaeoglobus fulgidus* archaeal complexes (31,40,44). We also demonstrate that THUMPD3 is responsible for the formation of m^2^G at tRNA position 6, confirming the findings of a recent study that also shows the enzyme installs m^2^G at position 7 of tRNA^Trp^ (52), Figure 2C, Supplementary Figures S3B–D and S4A–C). Lack of either TRMT11 or THUMPD3 have only minor phenotypic effects. However, the concomitant absence of both THUMPD3 and TRMT11 results in an almost complete loss of m^2^G in tRNAs (Figure 2C) and strongly affects the proliferation of the HCT116 cancer cell line (Figure 2D, Supplementary Figure S5D). Rationalizing this profound growth defect, we revealed that concomitant loss of THUMPD3 and TRMT11 leads to reduced monosome and polysome levels and impairs global translation (Figure 2G,H; Supplementary Figure S6E). Collectively, these results indicate that the absence of individual m^2^G modifications at positions 6, 7 or 10 of tRNAs is not detrimental for most of the investigated phenotypes, whereas the combined absence of these m^2^G modifications on one or several tRNAs is important. This suggests functional interdependency of the m^2^G modifications at these positions. Such synergies have also been observed between other tRNA modifications. Indeed, the deletion of individual, non-essential genes encoding yeast tRNA modification enzymes targeting regions outside the anticodon stem loop rarely leads to obvious phenotypes whereas double mutants, lacking two tRNA modification enzymes, often exhibit more pronounced growth defects (104,105). For example, *S. cerevisiae* strains lacking either the *TRM11* (yeast homolog of *TRMT11*) or *TRM1* genes grow as WT whereas the *trm11Δ/trm1Δ* double mutant exhibits a severe growth defect (40). With the methods used here, we observe no effect of the individual or concomitant absence of m^2^G_6/7/10_ on tRNA stability (Supplementary Figure S6A-C; Supplementary Table S7), folding (Figure 2E) or aminoacylation (Figure 2F). Although future studies will be required to dissect the precise molecular functions of these m^2^G modifications, our study highlights their combined importance for optimizing tRNA function during translation.

### THUMPD2 as the elusive MTase responsible for formation of m^2^G in the U6 snRNA

The remaining TRMT112 MTase interactor revealed by our BioID analysis, THUMPD2, has thus far remained functionally uncharacterized. Here, we demonstrate that THUMPD2 is necessary for the *in cellulo* formation of m^2^G in RNAs with size ranging from 90 to 200 nt and that the recombinant *Bt*TRMT112–THUMPD2 complex catalyzes the *in vitro* formation of m^2^G in human RNAs with the same size (Figure 3C–E). Interestingly, in the early 1980s, U6 snRNA extracted from rodent cells was shown to contain one m^2^G modification at position 72 but the enzyme responsible for this modification remained elusive (53,54). Our data reveal that THUMPD2 interacts directly and specifically with the U6 snRNA and is necessary for the formation of m^2^G in human U6 snRNA (Figure 4C, D), implying that it is the long-sought MTase responsible for the formation of m^2^G at position 72. While we cannot exclude that THUMPD2 also has other small RNA targets, it is likely that methylation of the U6 snRNAs represents a key function. Our data indicate no activity of *Bt*THUMPD2–TRMT112 on RNAs < 90 nt, which encompasses tRNAs (Figure 3C, D) and we show that THUMPD2 does not directly associate with other snRNAs, including the U6atac of the minor spliceosome (Figure 4C). A recent report suggests the presence of Trm11-dependent m^2^G in yeast mRNA (106), but in human cells, m^2^G has not been detected outside small non-coding RNAs (tRNAs and snRNAs). While it is possible that THUMPD2 contributes to methylation of other RNA species, the development of novel m^2^G detection and mapping approaches will be required to enable characterization of other potential sites and verify the MTases responsible for installing them.

The U6 snRNA, which base pairs with U2 and U4 snRNAs as well as pre-mRNAs, undergoes many structural remodeling events during biogenesis of the U6 snRNP and assembly of functional spliceosomes (96). Analysis of the THUMPD2 interactome did not reveal direct interactions with other snRNAs or strong association with known U6 biogenesis factors (Figure 4C, Supplementary Table 8, Supplementary Figure S8E) so the timing of m^2^G installation on U6 remains unknown. Currently, little is known about elements required for substrate recognition by the THUMPD2–TRMT112 complex. In the context of various tRNA modification enzymes, the THUMP domain interacts with the tRNA acceptor stem and senses the presence of the CCA tail (31,42,86,107), thus functioning as a molecular ruler directing the associated catalytic domain to target specific nucleotides within the tRNA acceptor stem/D-arm. In the future, it will be interesting to dissect the role of the THUMPD2 THUMP domain and determine if it is necessary for the U6 snRNA to adopt a (partial) tRNA-like structure to be modified by the THUMPD2–TRMT112 complex.

### m^2^G modification of the U6 snRNA is important for pre-mRNA splicing

The U6 snRNA plays a central role in pre-mRNA splicing by forming catalytic elements of the spliceosome active site (96). In particular, the phosphate group of U6–U_74_ plays a central role in splicing by coordinating the catalytic metal ions. Interestingly, the m^2^G_72_ nucleotide is located two nucleotides upstream of U_74_ and its phosphate group also coordinates one of the catalytic metal ions (Figure 4B). Compared to parental HCT116 cells, the proliferation of THUMPD2 KO1 and KO2 cells is strongly reduced (Figure 4E), likely due to alterations in pre-mRNA splicing. In line with our finding that the U6, but not the U6atac, snRNA is associated with THUMPD2 (Figure 4C), only 6% of genes that showed differential alternative splicing contained introns known to be targeted by the minor spliceosome (Olthof et al; https://doi.org/10.1101/2022.10.12.511939), which is not significant different from the genome average (chi-squared test *P*-value > 0.05).

mRNA-seq analyses revealed a plethora of alternative pre-mRNA splicing events affected in both the THUMPD2 KO1 and KO2 cells (Figure 5C, Supplementary Figure S9B–C, G–H). Closer analysis revealed that introns retained in cells lacking THUMPD2 are generally shorter, have shorter polypyrimidine tracts and lower splice site quality than average (Figure 5G, Supplementary Figure S9F). Furthermore, the expression levels of mRNAs with increased intron retention in the THUMPD2 KO1 cell line were significantly reduced compared to mRNAs with better intron removal (Figure 5H). This might be due to the presence of in-frame premature stop codons on retained introns, which then are likely to trigger the nonsense mediated mRNA decay quality-control pathway (108,109). As the absence of m^2^G on U6 snRNA does not affect its stability (Figure 4F), it remains to be understood precisely how this modification influences pre-mRNA splicing. It is possible that the G_72_ modification enhances the stability of the U6 snRNA internal stem-loop (ISL), thus rendering it important for splicing at sub-optimal sites. Interestingly, the effects observed on alternative splicing upon THUMPD2 depletion mirror those resulting from depletion of LARP7, which is important for several 2’-*O*-methylations present within U6 snRNA ISL (110,111). In human cells, depletion of TFIP11 also affects U6 snRNA 2’-*O*-methylation and results in decreased pre-mRNA splicing fidelity and increase in intron retention (112). Likewise, depletion of *Arabidopsis thaliana* FIO1, the orthologue of the METTL16 MTase responsible for installing m^6^A_43_ in the human U6 snRNA (113,114), affects pre-mRNA splicing efficiency and accuracy (115). The U6 snRNA epitranscriptomic marks are therefore emerging as key regulators of (alternative) pre-mRNA splicing.

## CONCLUSION

Using an unbiased approach, we have identified three additional MTases (TRMT11, THUMPD2 and THUMPD3) as direct partners of human TRMT112. We also demonstrate that these three TRMT112-MTase complexes act as holoenzymes to catalyze the formation of m^2^G in small RNAs. Both THUMPD3 and TRMT11 MTase subunits are essential for the introduction of m^2^G in tRNA body and are necessary for optimal protein synthesis and for cell proliferation. The THUMPD2 MTase introduces the m^2^G modification on U6 snRNA, the central RNA involved in the catalytic step of pre-mRNA splicing and is important for optimal pre-mRNA splicing. This study contributes to the characterization of the human enzymes responsible for ‘orphan’ RNA modifications. It also emphasizes on the central role of TRMT112 as an intrinsic component of holoenzymes modifying various RNAs to fine-tune two essential cellular processes.

## Supplementary Material

gkad487_Supplemental_Files

## Data Availability

The mRNA-seq datasets for the WT HTC116 and THUMPD2 KO cells are deposited in Gene Expression Omnibus (GEO) database [http://www.ncbi.nlm.nih.gov/geo/] under the accession code GSE219260. The mass spectrometry proteomics data from the BioID experiments have been deposited to the ProteomeXchange Consortium via the PRIDE (60) partner repository with the dataset identifier PXD038997. The mass spectrometry proteomics data from the THUMPD2 IP experiments have been deposited to the ProteomeXchange Consortium via the PRIDE (60) partner repository with the dataset identifier PXD038967 and 10.6019/PXD038967.
