# Correction to ‘N^2^-methylguanosine modifications on human tRNAs and snRNA U6 are important for cell proliferation, protein translation and pre-mRNA splicing’

**DOI:** 10.1093/nar/gkae524

**Published:** 2024-06-18

This is a correction to: Can Wang, Nathalie Ulryck, Lydia Herzel, Nicolas Pythoud, Nicole Kleiber, Vincent Guérineau, Vincent Jactel, Chloé Moritz, Markus T Bohnsack, Christine Carapito, David Touboul, Katherine E Bohnsack, Marc Graille, N2-methylguanosine modifications on human tRNAs and snRNA U6 are important for cell proliferation, protein translation and pre-mRNA splicing, *Nucleic Acids Research*, Volume 51, Issue 14, 11 August 2023, Pages 7496–7519, https://doi.org/10.1093/nar/gkad487

In the original publication of this article, there is an error in Figure 6 where the TRMT11 and THUMPD3 labels have been inverted.

The published article has been updated.

This change does not affect the results, discussion and conclusions presented in the article.

