# Supplementary material for: N 2-methylguanosine modifications on human tRNAs and snRNA U6 are important for cell proliferation, protein translation and pre-mRNA splicing
Source: Nucleic Acids Res. 2023 Jun 7;51(14):7496–519. doi: 10.1093/nar/gkad487 (PMC10415138; doi:10.1093/nar/gkad487)
Supplement: gkad487_Supplemental_Files [file gkad487_supplemental_files.zip › Wang-m2G-RNA-MTase-Sup-Final.pdf]

Supplementary data

N[2]-methylguanosine (m[2]G) modifications on human tRNAs and snRNA U6 are important for cell proliferation, protein translation and pre-mRNA splicing

Can Wang[1], Nathalie Ulryck[1], Lydia Herzel[2], Nicolas Pythoud[3], Nicole Kleiber[2], Vincent Guérineau[4], Vincent Jactel[5], Chloé Moritz[3], Markus T. Bohnsack[2,6], Christine Carapito[3], David Touboul[4,7], Katherine E. Bohnsack[2,*], Marc Graille[1,*]

[1]Laboratoire de Biologie Structurale de la Cellule (BIOC), CNRS, École polytechnique, Institut Polytechnique de Paris, 91120 Palaiseau, France
[2]Department of Molecular Biology, University Medical Center Göttingen, 37073 Göttingen, Germany
[3] Laboratoire de Spectrométrie de Masse BioOrganique, CNRS, Université de Strasbourg, IPHC UMR 7178, Infrastructure Nationale de Protéomique ProFI - FR2048, Strasbourg, France.
[4]Université Paris-Saclay, CNRS, Institut de Chimie des Substances Naturelles, UPR 2301, 91198, Gif-sur-Yvette, France
[5]Laboratoire de Synthèse Organique (LSO), CNRS, École polytechnique, ENSTA, Institut Polytechnique de Paris, 91120 Palaiseau, France
[6]Cluster of Excellence "Multiscale Bioimaging: from Molecular Machines to Networks of Excitable Cells" (MBExC), Göttingen, Germany
[7]Laboratoire de Chimie Moléculaire (LCM), CNRS, École polytechnique, Institut Polytechnique de Paris, 91120 Palaiseau, France

* Correspondence should be addressed to KEB (katherine.bohnsack@med.uni-goettingen.de) or MG (marc.graille@polytechnique.edu)

Supplementary Text

Mapping of the tRNA positions targeted by human TRMT11-TRMT112 and THUMPD3-TRMT112 complexes on E.coli tRNA$_i^{Met}$

To map the positions modified by TRMT11-TRMT112 and THUMPD3-TRMT112 on *E. coli* tRNA$_i^{Met}$, this tRNA was incubated with the recombinant human complexes and then digested by RNase A prior to MALDI-MS/MS experiments as previously described (1). We mainly focused on digested oligonucleotides $_9$GGAGC$_{13}$ and $_4$GGGGs^{4}U$_8$, which are methylated by TRMT11-TRMT112 and THUMPD3-TRMT112, respectively (Supplementary Figure S3A and S3D).

For the THUMPD3-TRMT112 complex, the d3 and w3 fragments from the $_4$GGGGs^{4}U$_8$ oligonucleotide were analyzed (Supplementary Figure S3D). The *m/z* value of the w3 fragment is shifted by 14 units from 1111.6 for the tRNA alone to 1125.7 in the presence of THUMPD3-TRMT112 complex, indicating that the mono-methylation does not occur at either G$_4$ or G$_5$ (Supplementary Figure S3B). Furthermore, the *m/z* value of the d3 fragment lacking one guanine base at position either G$_4$, G$_5$ or G$_6$, is also shifted by 14 unit upon incubation of the tRNA with the human THUMPD3-TRMT112 complex (from 903.5 to 917.4), demonstrating that the methylation catalyzed by THUMPD3-TRMT112 occurs at position 6 of the *E. coli* tRNA$_i^{Met}$ (Supplementary Figure S3C).

For the $_9$GGAGC$_{13}$ oligonucleotide obtained upon digestion of the tRNA alone, the fragments c2 and d2 lacking the guanine base from either G$_9$ or G$_{10}$ (*m/z* value of 540.1 and 558.1, respectively; Supplementary Figure S3D-E) were considered first. Upon incubation of the tRNA with TRMT11-TRMT112, the *m/z* values of both fragments are shifted by 14 units (554.2 for c2 and 572.2 for d2 fragments lacking on G base), meaning that one methyl group is added on either base G$_9$ or G$_{10}$. As in the presence of the TRMT11-TRMT112 complex, no *m/z* change is observed on fragment c1 containing the sole G$_9$ compared to the tRNA alone (Supplementary Figure S3D), we conclude that this enzyme adds a methyl group at position G$_{10}$ of *E.coli* tRNA$_i^{Met}$.

Depletion of TRMT11, THUMPD3 and THUMPD2 proteins by CRISPR-Cas9

To inactivate the expression of each of the MTases of interest, the CRISPR-Cas9 technique was used to generate DNA double-stranded breaks (DSBs) within the targeted gene region and thereby induce random mutation (insertion of nucleotides due to mispairing) through non-homologous end joining (NHEJ) pathway of DNA damage repair (2). For TRMT11 and THUMPD3 genes, two different sgRNAs were used whereas for THUMPD2, only one sgRNA was used (Supplementary Figures S5A-B and S7A; Supplementary Table S2). The resulting cells were screened for the lack of

expression of the different proteins of interest by western blot using antibodies directed against these proteins of interest (Supplementary Table S3). For cells not expressing the targeted protein, the presence and nature of the mutations were characterized by sequencing the targeted regions of the genes using oligonucleotides listed in Supplementary Table S2.

For TRMT11 KO cell lines, the sequencing results showed that in both cases, a T nucleotide was inserted exactly on the sgRNA-targeted sites, causing a frameshift event leading in the appearance of a premature stop codon (Supplementary Figure S5A). For the TRMT11 KO1 cell line, the insertion induced a frameshift in the Tyr377 codon associated with the emergence of a premature stop codon 6 codons downstream (p.Tyr377Leufs*6). Regarding the TRMT11 KO2 cell line, the insertion caused a frameshift in the Leu366 codon associated with the emergence of a stop codon 17 codons downstream (p.Leu366Phefs*17).

For THUMPD3, the sgRNAs designed to inactivate THUMPD3 expression bind to the last exon (E9) of *THUMPD3* gene (Supplementary Figure S5B). The sequencing of the targeted locus revealed a one nucleotide (A) insertion induced by both sgRNAs, resulting in frame-shifting events that generated a delayed stop codon (p.Val465Glyfs*58 for THUMPD3 KO1 and p.Asn471Lysfs*52 for THUMPD3 KO2).

Two TRMT11/THUMPD3 double KO1 and KO2 cell lines were generated by mutating the *THUMPD3* gene using sgRNA-MG1 or sgRNA-MG3 (Supplementary Table S2), respectively, in the TRMT11 KO2 cell line (Supplementary Figure S5C). The same mutations as those observed for the THUMPD3 KO1 and KO2 cell lines were obtained.

For THUMPD2 protein, we obtained two cell lines showing depletion in the protein of interest (Figure 3B). For the THUMPD2 KO1 cell line, the genomic DNA sequencing results revealed the insertion of an A exactly on the site targeted by sgRNA-MG7 on THUMPD2 exon 6 (Supplementary Figure S7A). This nucleotide insertion was also detected in the mRNA-seq data as more than 80% of the reads have an insertion at this position of THUMPD2 exon 6 in the THUMPD2 KO1 cell line (Supplementary Figure S7C). This resulted in a frameshift event causing the appearance of a premature stop codon 9 codons downstream the inserted nucleotide (p.Ala287Serfs*9; Supplementary Figure S7A). For the THUMPD2 KO2 cell line, genomic DNA sequencing revealed the presence of a three nucleotide insertion that encodes a stop codon (TAA) at the targeted site, which leads to expression of a truncated protein (p.Ala287*). The RNA-seq analysis showed intron retention around the targeted exon (Supplementary Figure S7B) and a marked coverage drop around the introduced stop codon (Supplementary Figure S7D).

Alternative splicing analysis

To assess alternative splicing (AS) changes, two alternative bioinformatics approaches were utilized: rMATS and FRASER. rMATS quantifies AS changes in an exon-centric way and provides inclusion values alternative 5′ splice sites (A5SS), alternative 3′ splice sites (A3SS), mutually exclusive exons (MXE), retained introns (RI), and for skipped exons (SE). For RI events, exon coordinates are given as output and thus it cannot be easily evaluated which introns are considered retained or better spliced. Hence, alternative splicing was also assessed with using the software package FRASER that quantifies intron retention and alternative splice site usage based on individual 3' and 5' splice sites (SS) (3). Psi3, psi5, theta3 and theta5 counts were obtained with FRASER for the individual replicates. Psi5 and psi3 correspond to the 'percent spliced in' of the respective 5' or 3' splice site. Theta5 and theta3 reflect intron retention computed at the 5' and 3' splice site, respectively. The outputs obtained from rMATS and FRASER together yielded consistent results. In both cases, intron retention was the most prevalent type of alternative splicing when considering the relative proportion of significantly changed events compared to all events that passed the minimal read cutoff criteria (Figure 5C, Supplementary Figure S9B,C). In absolute terms skipped exons were most abundant, but these are also the group with the most events passing the read cutoff (e.g. 58068 skipped exons versus 4421 introns in rMATS, Supplementary Figure S9G-H). The overlap of genes that were determined to have significantly more or less intron retention using both methods was highly significant as determined with the Chi-squared test. The respective p-values for more retained introns (up) and for less retained introns (down) are 2×10^{-26} and 4×10^{-35}. 56% of genes detected to have more intron retention in the THUMPD2 KO1 by rMATS also showed significantly more intron retention in the analyses performed using FRASER. Overall, > 10 times more events associated with altered intron retention were detected using FRASER than rMATS (Supplementary Figure S9G-H). Hence, the intron feature analysis is based on the FRASER dataset.

List of Supplementary tables (see .xlsx file)

Table S1: Plasmids used in this study

Table S2: Oligonucleotides used for molecular cloning/site-directed mutagenesis in this study.

Table S3: Antibodies used in the study

Table S4: Oligonucleotides used as northern blot probes or for RT-qPCR

Table S5: MS data of BioID experiments

Table S6: Oligomeric states of TRMT112-MTase complexes as determined by SEC-MALLS

Table S7: tRNA expression level data

Table S8: MS data of THUMPD2 IP

Table S9: Gene expression changes between WT and THUMPD2 KO1/KO2 based on DEseq2 analysis

Table S10: rMATS results for all significant AS events

Table S11: Significant intron retention events called with FRASER (theta)

Table S12: Significant ‘percent spliced in’ splice sites called with FRASER (psi)

Table S13: Validation of alternative splicing events by RT-PCR

Legends to Supplementary Figures

Supplementary Figure S1: Validation of the interaction between human TRMT112 and MTases

A. Human METTL5 co-purifies with wild-type TRMT112 but not with the TRMT112 T5R mutant. BirA*-HA-TRMT112 proteins were expressed in HEK293T cells and captured using an anti-HA antibody attached to agarose beads. Co-immunoprecipitated proteins were detected by western blot using the indicated antibodies. As a control, we used a cell line expressing only the BirA*-HA tag. Samples were treated with or without benzonase to degrade nucleic acids.

B. Optimization of the expression levels of the different BirA* proteins in HEK293T cells. For BirA*-HA and eGFP-BirA*-HA (pMG1127), 2 μg of the corresponding plasmids were mixed with 3 μg of empty plasmid for transfection. For TRMT112-WT-BirA*-HA and TRMT112-T5R-BirA*-HA, 5 μg of the corresponding plasmids (pMG891 or pMG906, respectively) were used for transfection. The expression levels were assessed by western blot using anti-HA antibodies.

C. TRMT112 co-purifies with either human THUMPD3 (upper panel) or THUMPD2 (lower panel). 3×Flag proteins were expressed in HEK293T cells and captured using an anti-Flag antibody attached to agarose beads. Co-immunoprecipitated proteins were detected by western blot using the indicated antibodies. As a control, we used a cell line expressing only the 3×Flag tag. Samples were treated with or without benzonase to degrade nucleic acids.

Supplementary Figure S2: Human TRMT11 and THUMPD3 proteins physically interact with TRMT112. Chromatograms resulting from SEC-MALLS analysis of the human TRMT112-TRMT11 (A) and TRMT112-THUMPD3 (B) complexes. For clarity, only the main refractive index peak (black curve, left y-axis) and the molecular weight distribution calculated from light scattering along this peak (red curve, right y-axis) are shown. Insets: SDS-PAGE analysis of the protein complexes used for these experiments.

Supplementary Figure S3: The TRMT11-TRMT112 and THUMPD3-TRMT112 complexes are m^2G_{10} and m^2G_6 tRNA methyltransferases.

A. Cloverleaf representation of *E. coli* $tRNA_i^{Met}$. RNase A cleavage sites of interest in this study are indicated by red arrows. Gs at positions 6, 7 and 10 are indicated in pink, purple and light green, respectively. 4, D, T and Ψ stand for 4-thiouridine, dihydrouridine, thymidine and pseudouridine, respectively.

B. *In vitro* enzymatic assays performed using recombinant human TRMT11-TRMT112 and THUMPD3-TRMT112 complexes and *E. coli* $tRNA_i^{Met}$ as substrate. Three replicates were performed and error bars represent standard deviation.

C. HPLC-MS elution profiles of *E. coli* $tRNA_i^{Met}$ digested into nucleosides after incubation with MTase buffer (no enzyme) or incubated with human TRMT11-TRMT112 or THUMPD3-TRMT112 complexes. Absorbance at 254 nm (left Y axis, black solid lines) and absolute intensity of *m/z* values of 298^+ (corresponding to the m^2G protonated ion; right Y axis, red solid lines) are plotted. Three replicates were performed and a representative image is shown.

D. MALDI-TOF MS spectrum of the $_4GGGGs^4U_8$ (*m/z* value of 1721.1) and$_9GGAGC_{13}$ (*m/z* value of 1688.1) oligonucleotides obtained from *E. coli* $tRNA_i^{Met}$ digestion after incubation with MTase buffer (top panel), human TRMT11-TRMT112 (middle panel) or THUMPD3-TRMT112 (bottom panel) complexes. Three replicates were performed and a representative image is shown.

Supplementary Figure S4: Mapping of the m^2G nucleosides deposited by human TRMT11-TRMT112 and THUMPD3-TRMT112 complexes on *E. coli* $tRNA_i^{Met}$

A. The w3- and d3-type fragment ions for the $_4GGGGs^4U_8$ oligonucleotide generated upon digestion of the *E. coli* $tRNA_i^{Met}$ by RNase A and discussed in panels B and C. This nomenclature is based on McLuckey *et al.* (4).
B. CID spectrum of the w3-type fragment ion observed in *E. coli* $tRNA_i^{Met}$ incubated with MTase buffer (upper panel) or human THUMPD3-TRMT112 complex (lower panel). The expected position of methylated ions is depicted by a red asterisk.
C. CID spectrum of the d3-type fragment ions lacking one G base observed in *E. coli* $tRNA_i^{Met}$ incubated with MTase buffer (upper panel) or human THUMPD3-TRMT112 complex. The expected position of methylated ions is depicted by a red asterisk.
D. The c- and d-type fragment ions for the $_9GGAGC_{13}$ oligonucleotide generated upon digestion of the *E. coli* $tRNA_i^{Met}$ by RNase A and discussed in panels E and F. This nomenclature is based on McLuckey *et al.* (4).
E. CID spectrum of the c2- and d2-type fragment ions lacking one G base observed in *E. coli* $tRNA_i^{Met}$ incubated with MTase buffer (upper panel) or human TRMT11-TRMT112 complex (lower panel). The expected position of methylated ions is depicted by a red asterisk.

F. CID spectrum of the c1-type fragment ion observed in *E. coli* $tRNA_i^{Met}$ incubated with MTase buffer (upper panel) or human TRMT11-TRMT112 complex. The expected position of methylated ions is depicted by a red asterisk.

Supplementary Figure S5: Validation of the disruption of the *TRMT11* and *THUMPD3* genes in different cell lines

A. Validation of the HCT116 TRMT11 KO cell lines. Schematic representation of the human TRMT11 gene with exons shown as blue squares and introns as red lines. The sgRNAs (sgRNA-MG14 and sgRNA-MG16) used and the region their target on TRMT11 gene are shown. Comparison of the sequencing chromatograms for the WT and TRMT11 KO1 or KO2 cell lines, showing the insertion of one T nucleotide in the TRMT11 gene in KO1 and KO2 cell lines. The table summarizes the sequence of the TRMT11 gene targeted region in WT and KO cell lines and the proteins generated by these mutations. ↓ : Predicted cleavage site. T : introduced nucleotide. * : stop codon. The amino acid sequences resulting from the frameshift events are in italics and underlined.

B. Validation of the HCT116 THUMPD3 KO cell lines. Schematic representation of the human THUMPD3 gene with exons shown as blue squares and introns as red lines. The sgRNAs (sgRNA-MG1 and sgRNA-MG3) used and the region their target on THUMPD3 gene are shown. Comparison of the sequencing chromatograms for the WT and THUMPD3 KO1 or KO2 cell lines, showing the insertion of one A nucleotide in the THUMPD3 gene in KO1 and KO2 cell lines. The table summarizes the sequence of the THUMPD3 gene targeted region in WT and KO cell lines and the proteins generated by these mutations. ↓ : Predicted cleavage site. A : introduced nucleotide. * : stop codon. The amino acid sequences resulting from the frameshift events are in italics and underlined.

C. Validation of the HCT116 TRMT11/THUMPD3 double KO cell lines. The sgRNAs (sgRNA-MG1 and sgRNA-MG3) targeting THUMPD3 gene were used to mutate this gene in the TRMT11 KO2 cell line. Comparison of the sequencing chromatograms for the THUMPD3 targeted locus in TRMT11 KO2 and TRMT11/THUMPD3 KO1 or KO2 cell lines, showing the insertion of one A nucleotide in the THUMPD3 gene in KO1 and KO2 cell lines.

D. Colony formation assay in soft agar: representative pictures. 1000 cells were seeded and cultured for 10 days before fixation and staining with crystal violet.

Supplementary Figure S6: The absence of m^2G_6 and m^2G_{10} does not affect tRNA stability or folding.

A-C. Scatter plots comparing the difference in abundance (determined as $2^{-\Delta Ct}$) of tRNAs between TRMT11 KO2 (A), THUMPD3 KO1 (B) or TRMT11/THUMPD3 KO1 (C) cell lines and parental cell line. The mitochondrial tRNAs are shown in grey. Color code for cytoplasmic tRNAs absence of m^2G (black), experimentally confirmed presence of m^2G_6 (green), m^2G_{10} (red) or both (orange). The black line corresponds to fold changes in $2^{-\Delta Ct}$ of 1 (no difference). The blue lines correspond to fold changes in $2^{-\Delta Ct}$ lower than 0.5 or higher than 2.

D. Total RNAs extracted from HEK293 cell lines lacking METTL8 (METTL8 KO1 and KO2; (5)) were separated by native polyacrylamide gel electrophoresis alongside RNA extracted from wild-type (WT) HCT116 cells or those lacking the indicated m^2G MTases. Selected mitochondrial (mt-) and cytosolic tRNAs were detected by northern blotting. The presence and position of m^2G in the detected tRNAs is noted on the right. Two biologically independent experiments were performed and a representative image is shown.

E. Polysome profile analyses of the different cell lines. For each cell line, a representative curve has been chosen among five biological replicates.

Supplementary Figure S7: Validation of the disruption of the *THUMPD2* gene in HCT116 cells

A. Validation of the HCT116 THUMPD2 KO1 and KO2 cell lines. Schematic representation of the human THUMPD2 gene with exons shown as blue squares and introns as red lines. The sgRNA-MG7 used and the targeted region on THUMPD2 gene is shown. Comparison of the sequencing chromatograms for the WT and THUMPD2 KO1 or KO2 cell lines, showing the insertion in the *THUMPD2* gene of one A nucleotide in KO1 cell line and three nucleotides (TAA) in the KO2 cell line. The table summarizes the sequence of the *THUMPD2* gene targeted region in WT and KO1/KO2 cell lines and the proteins generated in each case. The amino acid sequences resulting from the frameshift events are in italics and underlined. The stop codon introduced in the THUMPD2 KO2 cell line is marked by a red asterisk.

B. Pooled read coverage profiles for wild-type and both knock-outs across the negative strand gene *THUMPD2*. Intron retention is observed especially in knock-out 2 around the target site of the sgRNA on exon 6.

C. Validation of single nucleotide insertion in KO1 from mapped mRNA-seq reads, allowing two mismatches. Error bars in barplot correspond to the standard deviation among the three replicates.

D. Pooled read coverage across exon 6 of THUMPD2 for wild-type and both knock-outs. Knock-out 2 shows a coverage drop around the region where the stop codon was inserted.

Supplementary Figure S8: Purification of recombinant TRMT112-THUMPD2 and fractionation of small RNAs for *in vitro* methylation assays.

A. Volcano plot showing that TRMT112 is the only protein significantly enriched by co-immunoprecipitation of human 3×Flag-THUMPD2 and detected by mass spectrometry.

B. Chromatogram resulting from the SEC-MALLS analysis of the bovine TRMT112-THUMPD2 complex. For clarity, only the main refractive index peak (black curve, left y-axis) and the molecular weight distribution calculated from light scattering along this peak (red curve, right y-axis) are shown. Inset: SDS-PAGE analysis of the protein complex used for these experiments.

C. SEC chromatogram of total tRNAs purified from parental or THUMPD2 KO1 HCT116 cell lines.

D. 8M acryl-urea gel of the various RNA populations and identification of fractions pooled to generate the large rRNA (blue box), 90-200 nt RNAs (green box) and <90 nt tRNAs (orange box) used in experiments presented in Figure 4C-D.

E. IP of Flag-His tagged THUMPD2 to search for U6 biogenesis factors. Inputs and eluates of anti-Flag immunoprecipitation experiments of Flag-His-THUMPD2, THUMPD2-His-Flag or the tag alone were analyzed by western blotting using the indicated antibodies.

Supplementary Figure S9: THUMPD2-dependent mRNA expression and splicing analysis

A. Heat-map of Pearson correlation coefficients of mRNA-seq gene expression for three replicates per condition.

B. Fraction of significant alternative splicing events identified with the splice-site centric method FRASER for KO1. Psi5 and psi3 correspond to the ‘percent spliced in’ of the respective 5’ or 3’ splice site. Theta5 and theta3 reflect intron retention computed at the 5’ and 3’ splice site, respectively. 95% confidence intervals are given as error bars (bootstrapping, n = 100). Dark bars reflect the overlap with splicing changes in the same direction for the THUMPD2 KO2 cell line.

C. Fraction of significant alternative splicing events identified with the splice-site centric method FRASER for KO2. See B for details on labels, error bars and dark shading.

D. Intron features associated with psi3. No significant changes were detected with the Wilcoxon rank-sum test between the up and down groups. The dashed line corresponds to the median value of the group containing not significantly different psi3 events.

E. Intron features associated with psi3 as heatmap (same data as in D). Heatmaps show 1) the change of the median in introns with 3'SSs that are more (up) or less (down) included in KO1 (plotted as modified Z-score) relative to the median in introns without significant changes and 2) the significance of the changes plotted as -log10(p-value) from the Wilcoxon rank-sum test.

F. Intron feature distributions for more (up) or less (down) retained introns identified with FRASER (theta3). Extended data matching Figure 6G. Significance was assessed with the Wilcoxon rank-sum test ((*) $P < 0.05$, (**) $P < 0.01$). The dashed line reflects the median value of the group containing not significantly different theta3 events.

G. Absolute numbers of significant alternative splicing events identified with rMATS and FRASER for THUMPD2 KO1. Significance of the overlap in genes with intron retention events was tested with the Chi-squared test (p-values are 2×10^{-26} for more retained introns (up) and 4×10^{-35} for less retained introns (down)). Dark bars reflect the overlap with splicing changes in the same direction for the THUMPD2 KO2 cell line.

H. Absolute numbers of significant alternative splicing events identified with rMATS and FRASER for THUMPD2 KO2. Dark bars reflect the overlap with splicing changes in the same direction for the THUMPD2 KO1 cell line. KO – knock-out, n.s. – not significant, PY – polypyrimidine, SS – splice site.

I. Intron overlap with snoRNAs. Intron-encoded snoRNAs overlap preferentially with retained introns in THUMPD2 KO1. 95% confidence intervals are given as error bars (bootstrapping, n = 100).

References

1. Wang, C., van Tran, N., Jactel, V., Guerineau, V. and Graille, M. (2020) Structural and functional insights into Archaeoglobus fulgidus m2G10 tRNA methyltransferase Trm11 and its Trm112 activator. *Nucleic Acids Res*, **48**, 11068-11082.

2. Ran, F.A., Hsu, P.D., Wright, J., Agarwala, V., Scott, D.A. and Zhang, F. (2013) Genome engineering using the CRISPR-Cas9 system. *Nat Protoc*, **8**, 2281-2308.

3. Mertes, C., Scheller, I.F., Yepez, V.A., Celik, M.H., Liang, Y., Kremer, L.S., Gusic, M., Prokisch, H. and Gagneur, J. (2021) Detection of aberrant splicing events in RNA-seq data using FRASER. *Nat Commun*, **12**, 529.

4. McLuckey, S.A., Van Berkel, G.J. and Glish, G.L. (1992) Tandem mass spectrometry of small, multiply charged oligonucleotides. *J Am Soc Mass Spectrom*, **3**, 60-70.

5. Kleiber, N., Lemus-Diaz, N., Stiller, C., Heinrichs, M., Mai, M.M., Hackert, P., Richter-Dennerlein, R., Hobartner, C., Bohnsack, K.E. and Bohnsack, M.T. (2022) The RNA methyltransferase METTL8 installs m(3)C32 in mitochondrial tRNAs(Thr/Ser(UCN)) to optimise tRNA structure and mitochondrial translation. *Nat Commun*, **13**, 209.

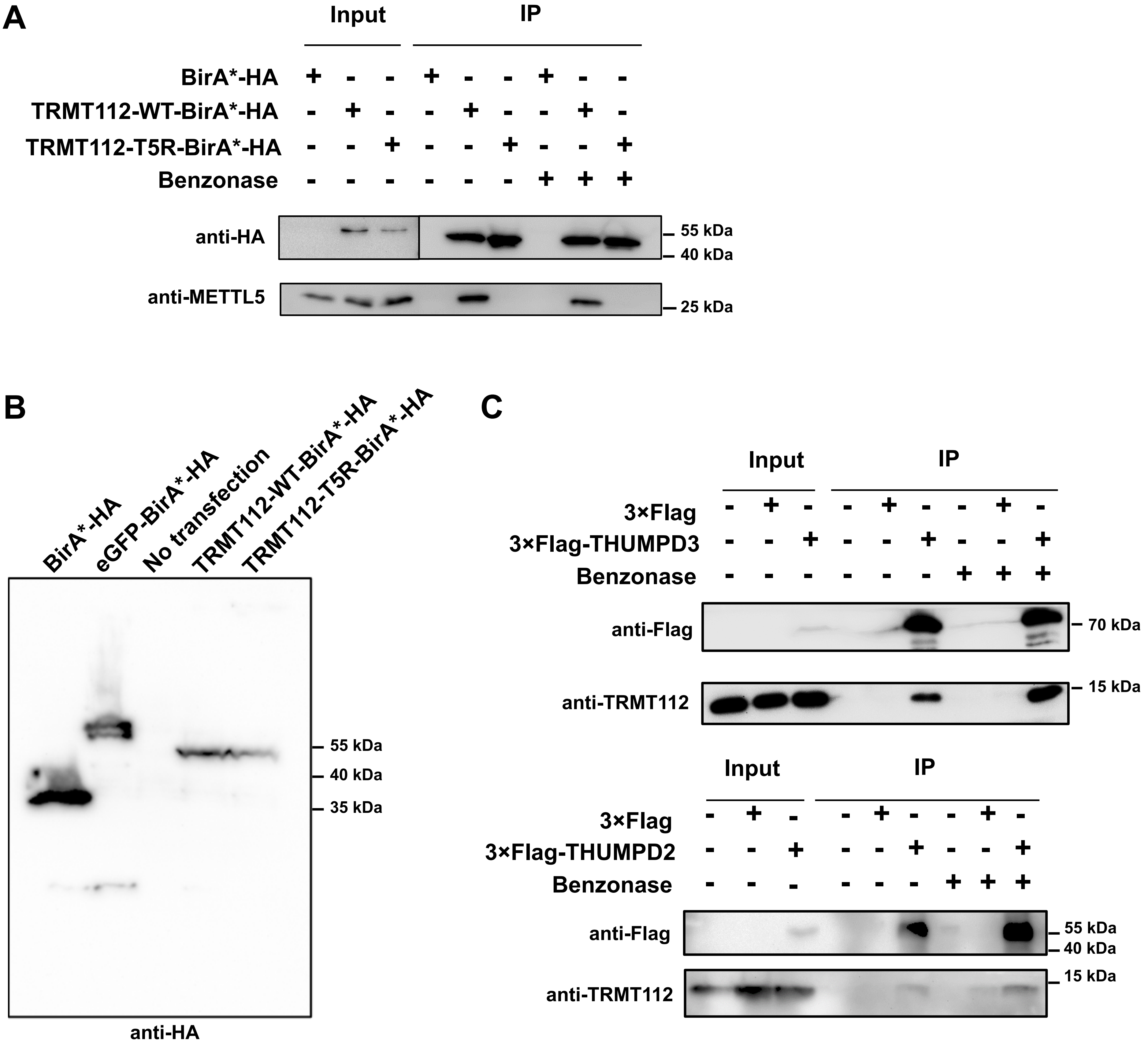

Supplementary Figure S1

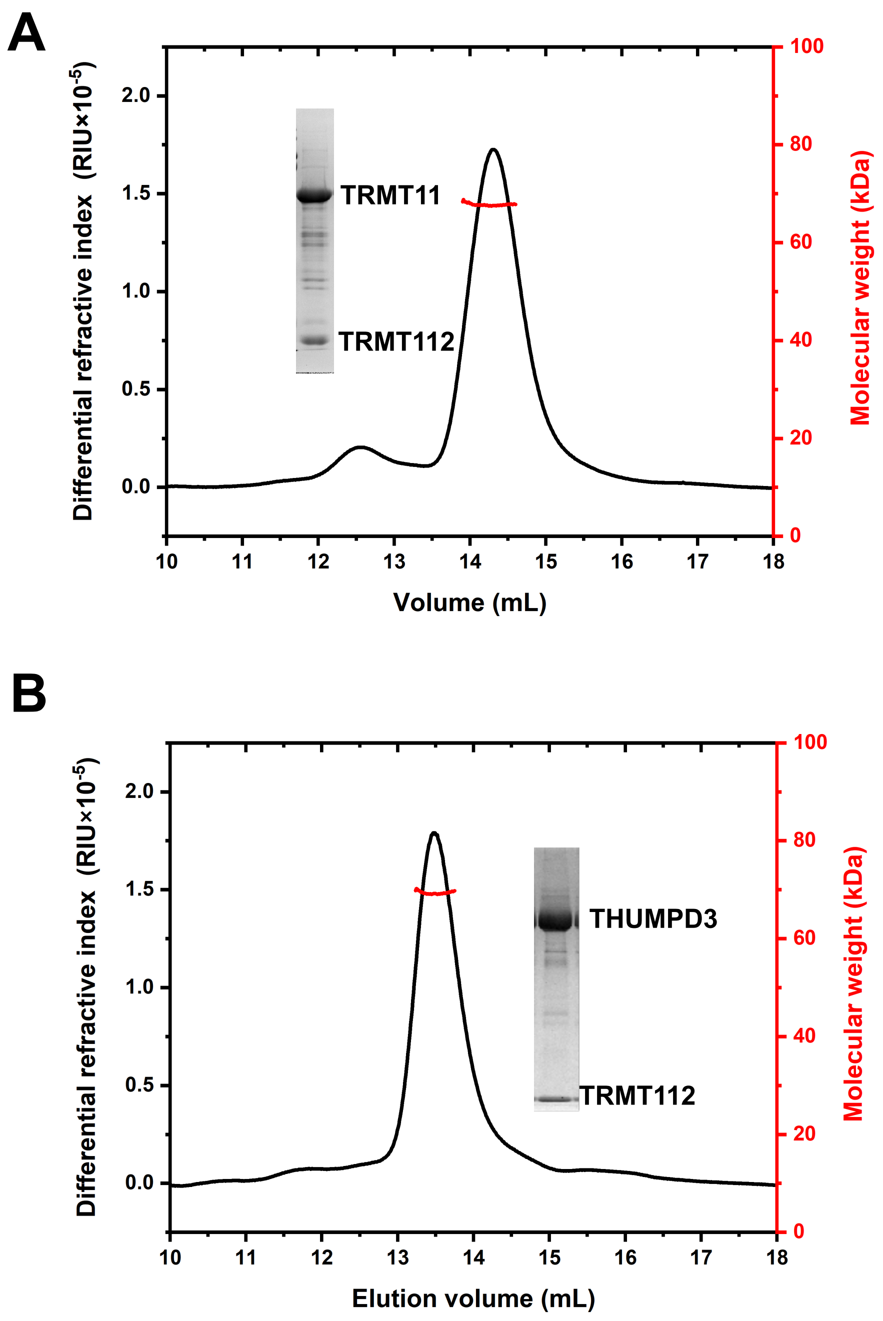

Supplementary Figure S2

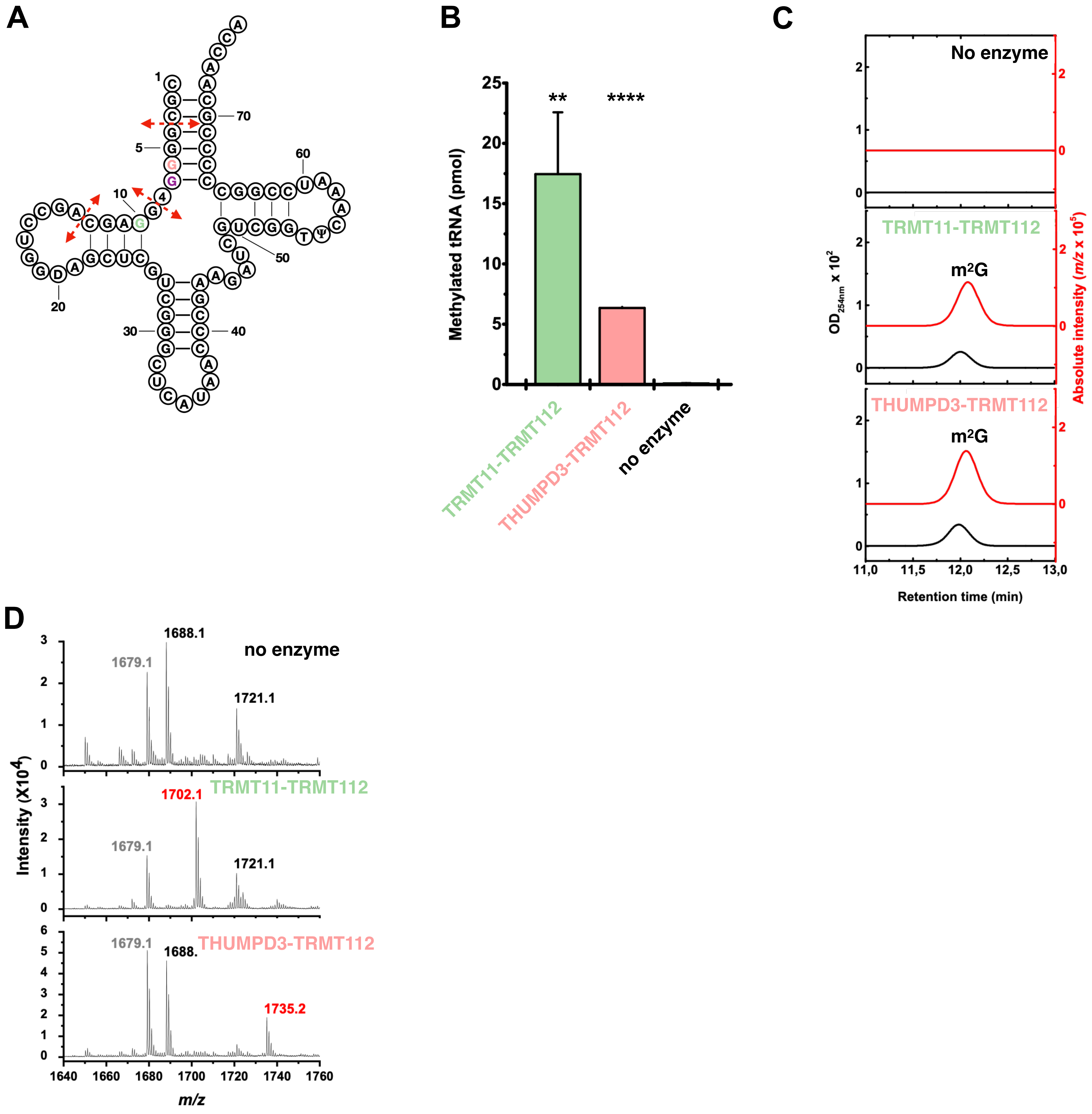

Supplementary Figure S3

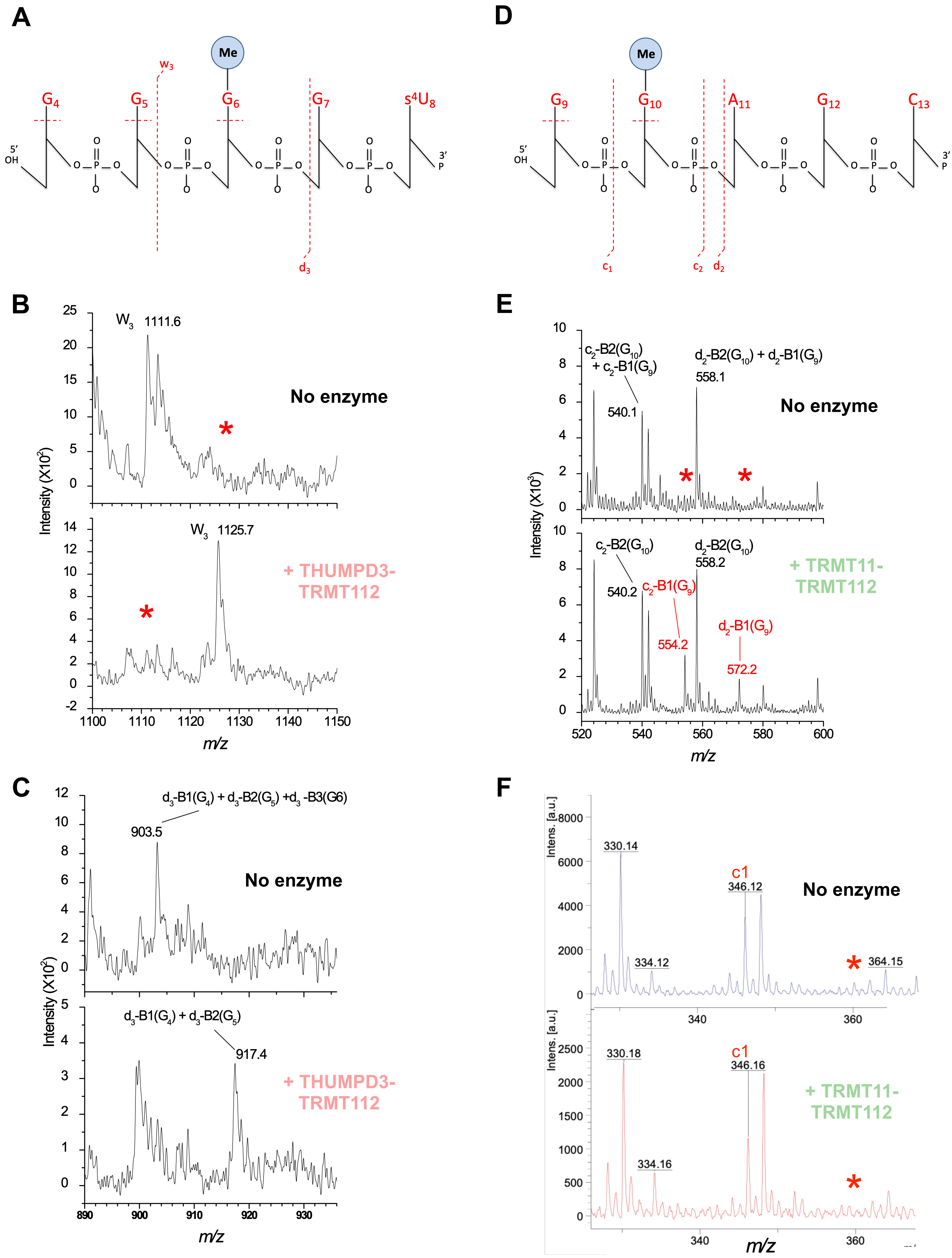

Supplementary Figure S4

A TRMT11 KO

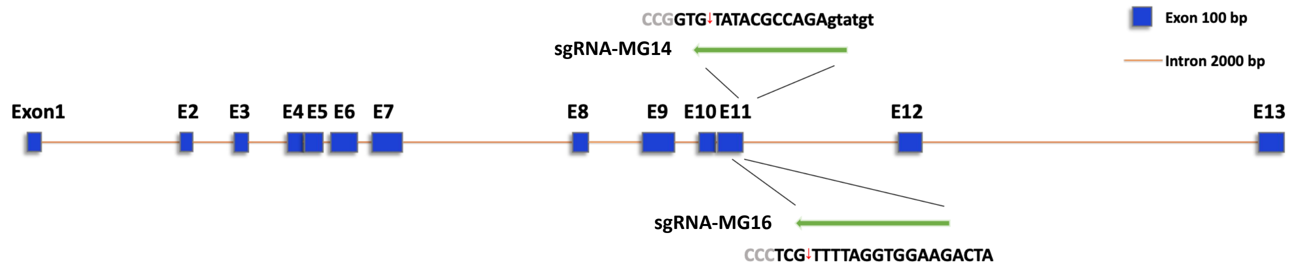

Cell line	sgRNA	Genome sequence	Amino acid sequence	Effect
WT		GTG↓TATACGCCAGA	^{376}VYTPEYTE^{383}	-
TRMT11 KO1	sgRNA-MG14	GTGTTATACGCCAGA	^{376}V*LYARIH**^{383}	Premature stop codon
WT		TCG↓TTTTAGGTGGAAGACTA	^{365}VLGGRLVYWLPVYTPEYTE^{383}	-
TRMT11 KO2	sgRNA-MG16	TCGTTTTTAGGTGGAAGACTA	^{365}V*FRWKTSLLVTGYARIH**^{383}	Premature stop codon

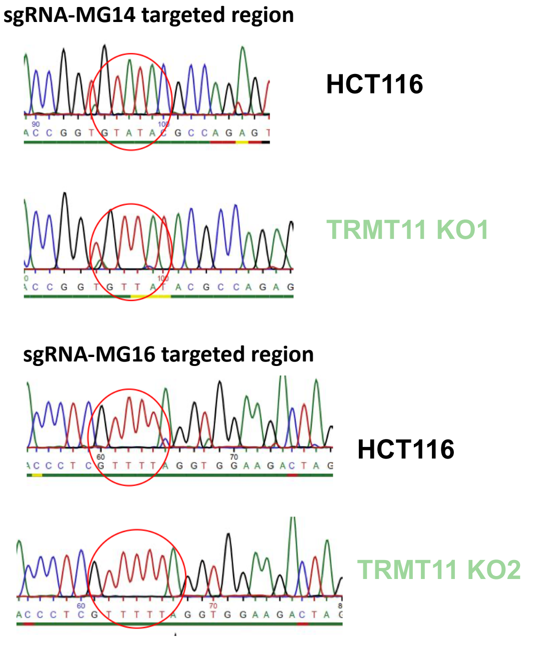

B THUMPD3 KO

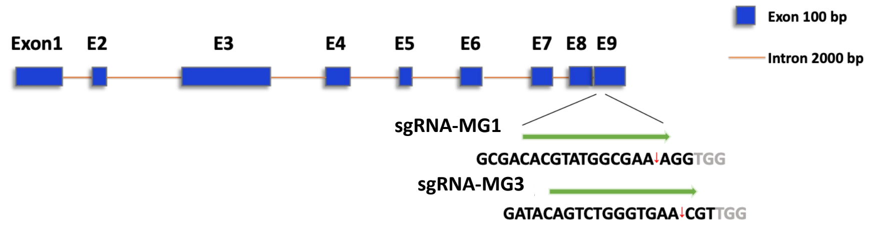

Cell line	sgRNA	Genome sequence	Amino acid sequence	Effect
WT		GCAACACGTATGGCGAA↓AGG	^{464}KCDTVW…LWQCKE*^{508}	-
THUMPD3 KO1	sgRNA-MG1	GCAACACGTATGGCGAAAAGG	^{464}K*GGYSLG…SHHWKC**^{522}	Delayed stop codon
WT		GATACAGTCTGGGTGAA↓CGT	^{470}VNVGGL…LWQCKE*^{508}	-
THUMPD3 KO2	sgRNA-MG3	GATACAGTCTGGGTGAAACGT	^{365}V*KRWWSS…SHHWKC**^{522}	Delayed stop codon

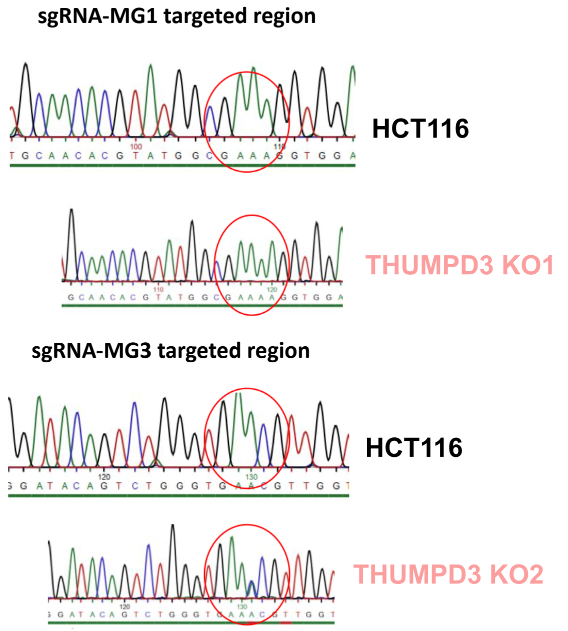

C TRMT11/THUMPD3 KO

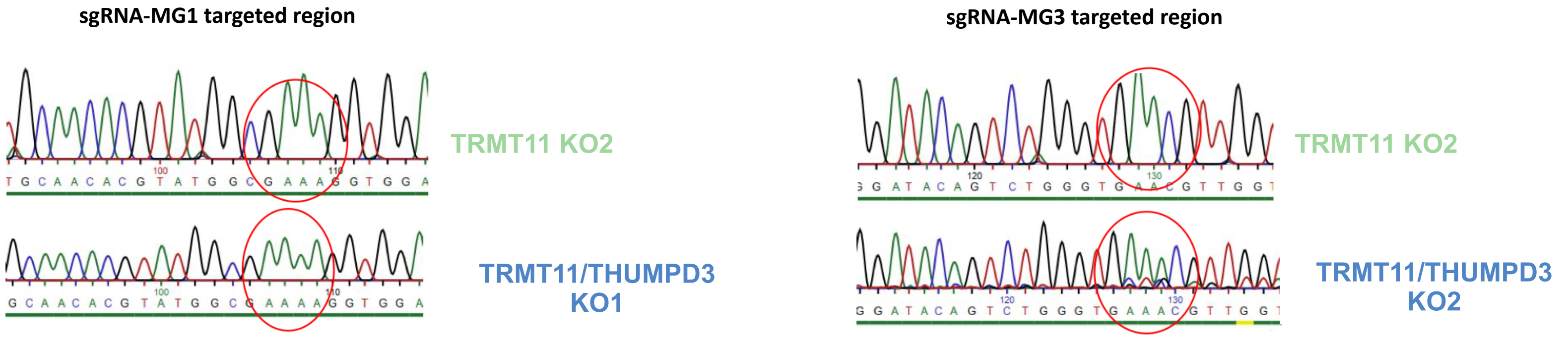

D

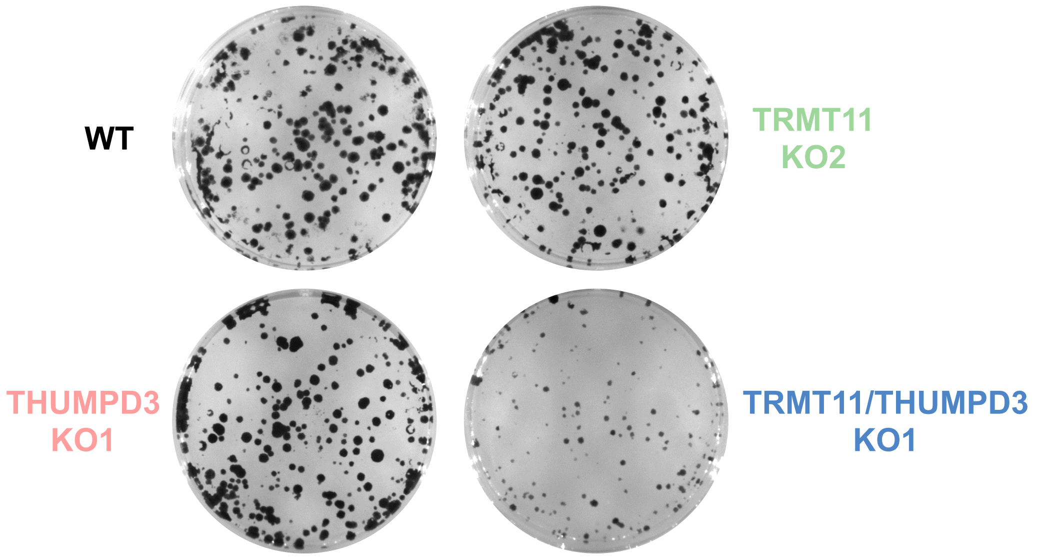

Supplementary Figure S5

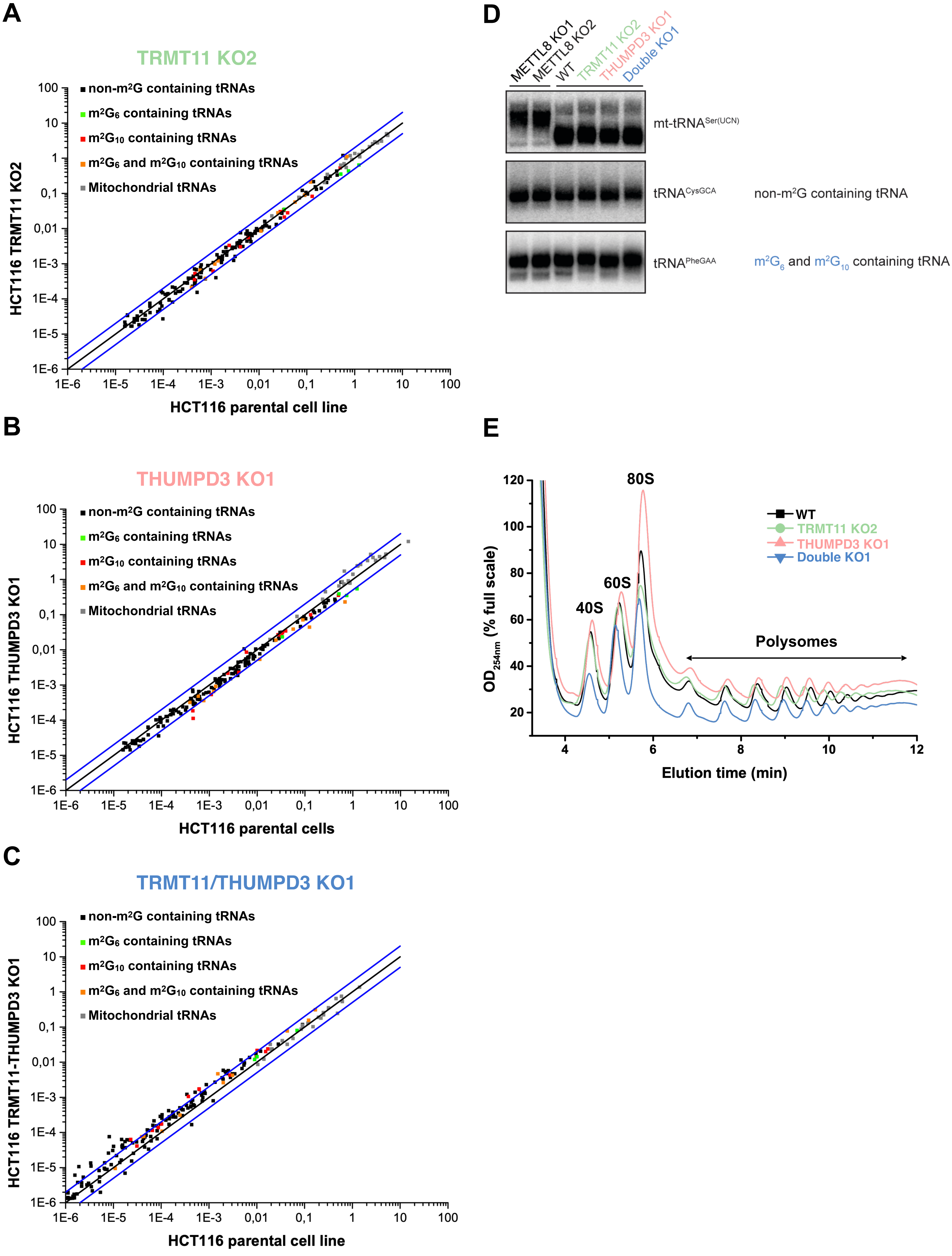

Supplementary Figure S6

A THUMPD2 KO1

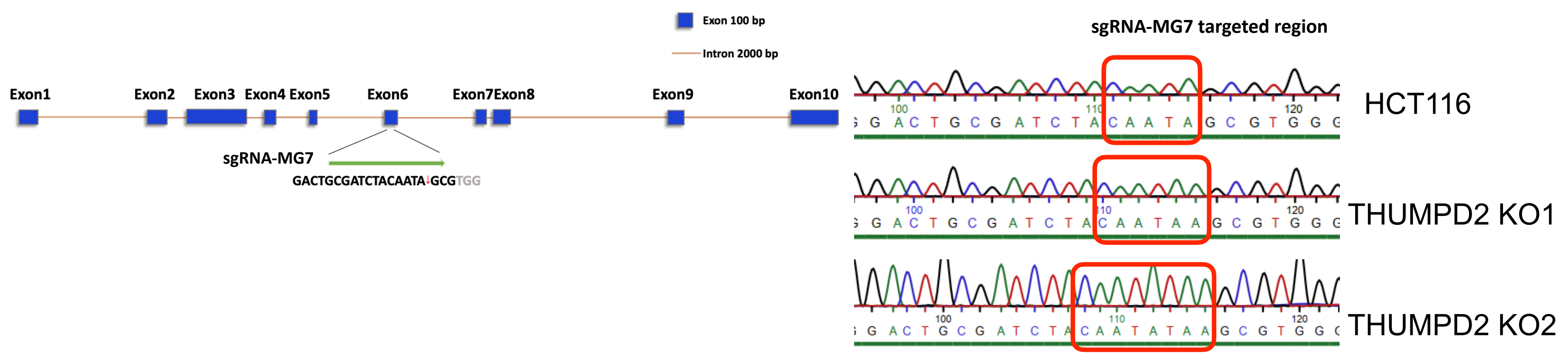

Cell line	sgRNA	Genome sequence	Amino acid sequence	Effect
WT		GACTGCGATCTACAATA↓GCG	^{281}GLRSTIAWAMASLAD^{295}	-
THUMPD2 KO1	sgRNA-MG7	GACTGCGATCTACAATA**A**GCG	^{281}GLRSTI*SVGNGISG**^{295}	Premature stop codon
THUMPD2 KO2	sgRNA-MG7	GACTGCGATCTACAATA**TAA**GCG	^{281}GLRSTI*^{287}	Premature stop codon

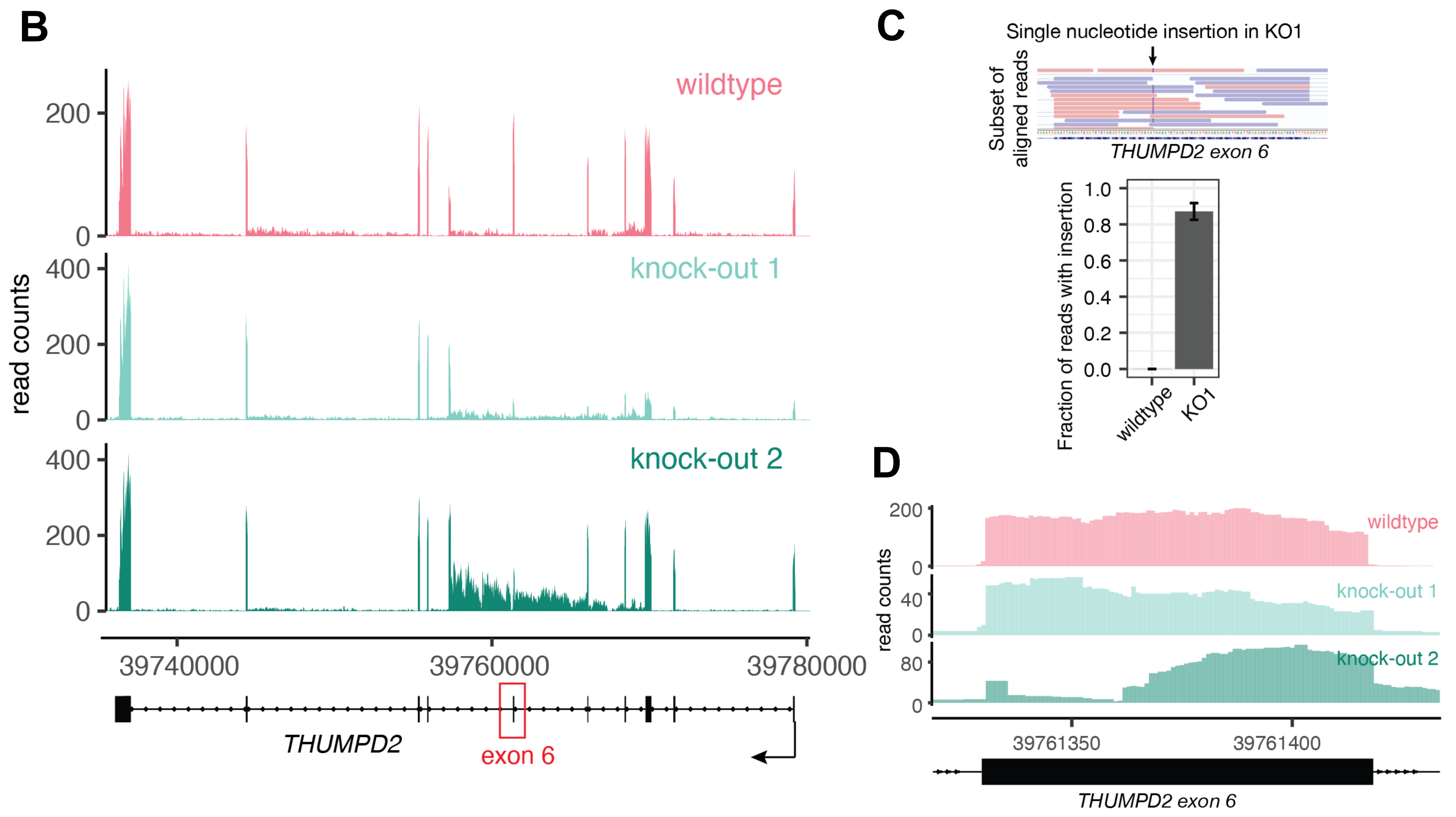

Supplementary Figure S7

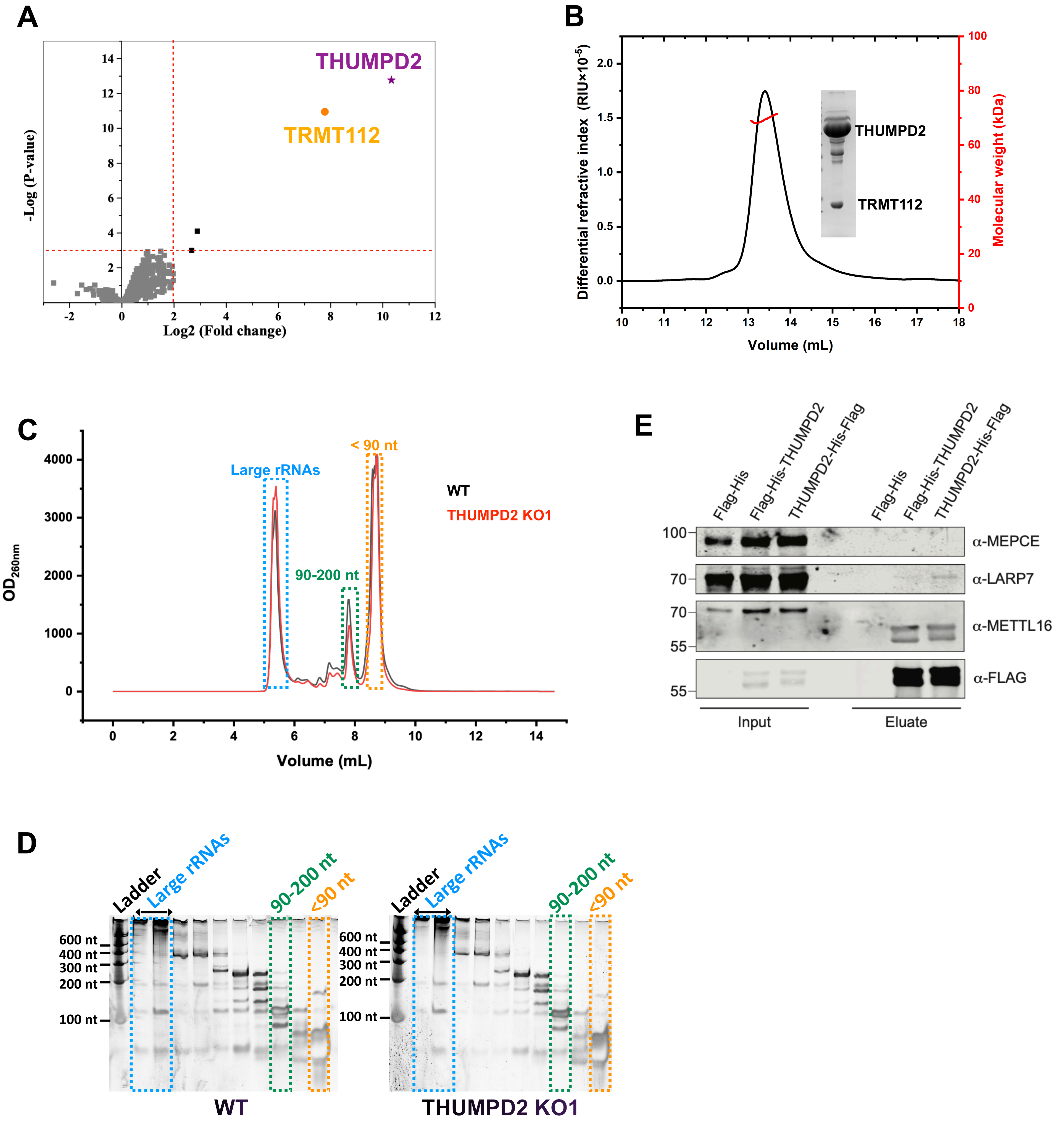

Supplementary Figure S8

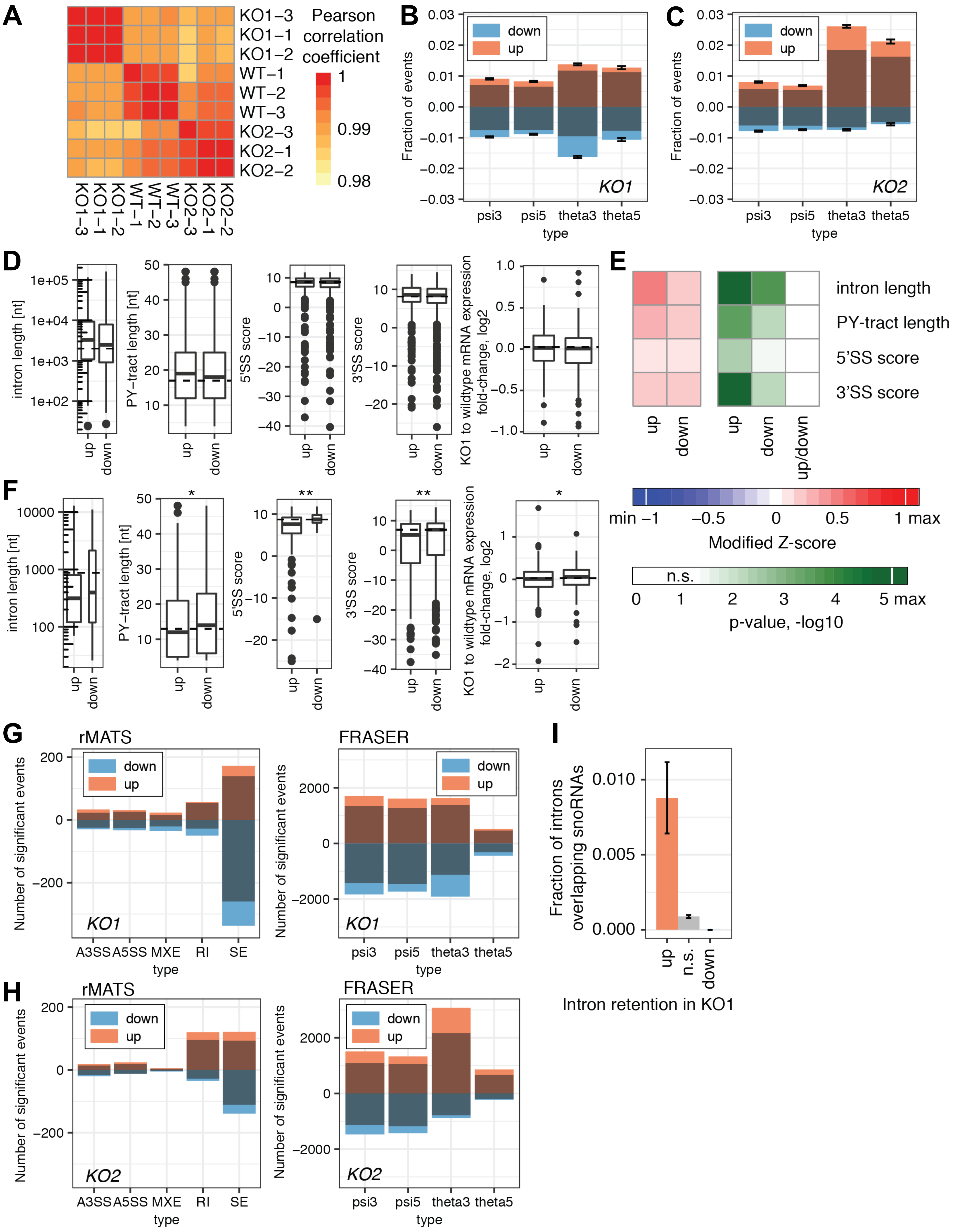

Supplementary Figure S9
